# Machine learning-assisted prognostic model for mortality in ICU patients with culture-confirmed *Klebsiella pneumoniae* infection: a preliminary single-center retrospective cohort study

**DOI:** 10.3389/fcimb.2026.1858732

**Published:** 2026-06-19

**Authors:** Xu Ran, Zhaojun Wang, Jingjing Shao, Ziyue Ma, Qinfu Liu, Dan Yang, Gang Li, Xiaojun Yang

**Affiliations:** 1 The First School of Clinical Medicine, Ningxia Medical University, Yinchuan, China; 2 Department of Critical Care Medicine, General Hospital of Ningxia Medical University, Yinchuan, China; 3 Medical Experimental Center, General Hospital of Ningxia Medical University, Yinchuan, China; 4 Ningxia Key Laboratory of Clinical and Pathogenic Microbiology, General Hospital of Ningxia Medical University, Yinchuan, China

**Keywords:** intensive care unit, *Klebsiella pneumoniae*, machine learning, mortality, prognostic model

## Abstract

**Background:**

*Klebsiella pneumoniae* (KP) is a major pathogen in intensive care units and is associated with substantial mortality. However, prognostic tools specifically developed for intensive care unit (ICU) patients with KP infection remain limited. This study aimed to identify stable prognostic factors and develop a predictive model for mortality in this population.

**Methods:**

A total of 587 adult ICU patients with culture-confirmed KP infection (first positive culture) at a tertiary hospital between August 2020 and February 2025 were retrospectively included. Candidate predictors were screened using least absolute shrinkage and selection operator (LASSO) and bootstrap-based stability selection to identify stable prognostic predictors. Using the final stable predictors, 11 models (including logistic regression and tree-based approaches) with 10-fold cross-validation were developed and compared. Model performance was evaluated using discrimination, calibration, and clinical utility. Generalized additive models (GAMs) were used to explore potential non-linear predictor–outcome associations.

**Results:**

Mortality occurred in 122/587 patients (20.8%). Five stable prognostic factors were identified: Acute Physiology and Chronic Health Evaluation II (APACHE II) score, lactate, viral co-infection, acute kidney injury (AKI), and the alveolar–arterial oxygen gradient (A-aDO_2_). Among the evaluated algorithms, logistic regression achieved the most favorable balance of discrimination, calibration, and interpretability, with a cross-validated area under the receiver operating characteristic curve (AUC) of 0.809. Non-linear or threshold-like patterns were observed for APACHE II (approximately 16.56), lactate (approximately 2.87 mmol/L), and the A-aDO_2_ (approximately 203.27 mmHg). Based on logistic regression, a nomogram and a web-based calculator were constructed for individualized risk estimation.

**Conclusion:**

A parsimonious model based on five stable variables showed good performance for predicting mortality among ICU patients with KP infection. However, these findings are exploratory and require external validation before any clinical application.

## Introduction

*Klebsiella pneumoniae* (KP) is a common Gram-negative opportunistic pathogen and a persistent public health threat (Li et al., 2025; Sati et al., 2025). It is a leading cause of hospital-acquired infections in the intensive care unit (ICU) (e.g., ventilator-associated pneumonia, bloodstream infections, and intra-abdominal infections) and an important agent of community-acquired invasive infections (Chen et al., 2023; Elbehiry et al., 2026). According to the 2019 Global Burden of Disease study, KP infections caused approximately 790,000 deaths, with an age-standardized mortality rate of 10.6 per 100,000, ranking among the top bacterial causes of death (Song et al., 2025). Data from China’s National Antimicrobial Resistance Monitoring Network show a steady increase in clinical isolation rates, reaching 21.3% in 2024 (Hu et al., 2025; Qin et al., 2024; Antimicrobial resistance profile of clinical isolates in hospitals across China: report from the CHINET Antimicrobial Resistance Surveillance Program, 2024, 2025).

KP infections present a broad clinical spectrum, from localized pneumonia and urinary tract infections to life-threatening sepsis, liver abscesses, and disseminated disease (Siu et al., 2012; Choby et al., 2020; Li et al., 2021). In ICU patients with multiple comorbidities and invasive procedures, KP often progresses to septic shock and multiple organ dysfunction syndrome (MODS), with mortality rates of 40%–50% (Zhang et al., 2020; Hafiz et al., 2023; Teo et al., 2025). Prognosis is influenced by pathogen factors (virulence and resistance), host factors (age and comorbidities), infection characteristics, and timeliness of intervention (Kurt et al., 2024; Dai et al., 2021; Qiu et al., 2025). Current clinical assessment relies on generic scoring systems such as Acute Physiology and Chronic Health Evaluation II (APACHE II) and Sequential Organ Failure Assessment (SOFA), which are not tailored to bacterial infections and may not capture KP-specific pathophysiology. Precision medicine has promoted data-driven prognostic models, and machine learning offers advantages in handling non-linear relationships and complex interactions for infectious disease prediction (Luo et al., 2020; Elengickal et al., 2026). However, high-performance prognostic models specifically for KP infection, particularly in high-risk ICU populations, remain limited (Li et al., 2023). Existing studies are often small-sample, use traditional statistical methods, and lack systematic comparisons of modern machine learning algorithms (Guan et al., 2025).

Therefore, we retrospectively analyzed ICU patients with KP infection at Ningxia Medical University General Hospital to 1) identify predictors of mortality using least absolute shrinkage and selection operator (LASSO) regression, 2) develop and internally validate prognostic models combining traditional regression with machine learning algorithms, 3) explore non-linear relationships and threshold effects of key continuous variables via GAM, and 4) establish a clinically interpretable tool for early risk stratification.

## Methods

### Study design and population

This study selected 587 adult inpatients with *K. pneumoniae* identified through microbiological cultures at the ICU of Ningxia Medical University General Hospital from August 2020 to February 2025, including only those with the first positive culture. Based on the survival status during hospitalization, the subjects were divided into a death group (122 cases) and a survival group (465 cases).

Inclusion criteria were as follows: 1) age ≥ 18 years, 2) the pathogen is a pathogenic bacterium (excluding colonization or contamination), and 3) specimens must be qualified and clinical data must be complete.

Exclusion criteria were as follows: 1) age < 18 years, 2) colonization or contamination, and 3) outpatient cases or missing key data.

This study was approved by the Medical Research Ethics Committee of the General Hospital of Ningxia Medical University (Approval No. KYLL-2024-1425). As it was a retrospective observational study, the requirement for informed consent was waived.

### Diagnostic criteria

KP infection presented with clinical signs (fever, hypothermia, leukocytosis, hypotension, purulent secretions, or radiographic infiltrates) plus positive culture from a normally sterile site, or significant growth from a non-sterile site (bronchoalveolar lavage (BAL) ≥ 10^4^ CFU/mL and endotracheal aspirate ≥10^5^ CFU/mL) (Kalil et al., 2016). Colonization was indicated by positive culture without clinical signs or anti-KP therapy (Rossi et al., 2021). These were adjudicated by two physicians.

Acute kidney injury (AKI)—Kidney Disease: Improving Global Outcomes (KDIGO) 2012 indicated serum creatinine rise ≥0.3 mg/dL within 48 h, or ≥1.5× baseline within 7 days, or urine output <0.5 mL·kg^−1^·h^−1^ for 6 h (Khwaja, 2012).

Viral co-infection was defined as a positive PCR result (FilmArray RP2.1 or equivalent) from respiratory or blood samples collected within 48 hours of the first KP-positive culture. The panel routinely included influenza A/B, respiratory syncytial virus (RSV), adenovirus, human metapneumovirus (hMPV), rhinovirus/enterovirus, parainfluenza types 1–4, and SARS-CoV-2. Cytomegalovirus (CMV) and Epstein–Barr virus (EBV) were tested only when clinically indicated (e.g., unexplained fever, lymphocytosis, or organ dysfunction) (Ranadheera et al., 2022). Viral testing was not performed on all patients. It was ordered at the discretion of the treating physician based on clinical suspicion.

The alveolar–arterial oxygen gradient (A-aDO_2_) was calculated as [FiO_2_ × (Patm − PH_2_O) − PaCO_2_/RQ] − PaO_2_, where Patm = 655 mmHg (local barometric pressure at Yinchuan altitude of approximately 1,100 m), PH_2_O = 47 mmHg, and RQ = 0.8, with only arterial blood gases on known, stable FiO_2_ on the day of KP diagnosis or within 24 h prior (Knaus et al., 1985).

### Data collection

Data were extracted from the hospital information system, including the following: general information [gender, age, and body mass index (BMI)], underlying diseases (diabetes, hypertension, cardiovascular diseases, chronic respiratory diseases, liver diseases, biliary diseases, chronic kidney disease, malignant tumors, and immunosuppression), infection characteristics (community/hospital-acquired, site of infection, sepsis, migratory infections, concurrent fungal/viral infections, multidrug resistance, and carbapenem resistance), laboratory indicators (complete blood count, arterial blood gas, liver and kidney function, cardiac markers, coagulation function, and inflammatory markers), invasive procedures (mechanical ventilation, central venous catheterization, and catheterization), therapeutic interventions (vasopressor drugs, hormone use, and history of abdominal surgery), severity scoring [APACHE II, SOFA, and Glasgow Coma Scale (GCS)], and outcomes (mortality rate). Laboratory indicators were based on the first test values obtained on the day of infection diagnosis or upon admission.

### Laboratory methods

Bacterial isolation and culture, as well as drug susceptibility testing, were conducted according to standard operating procedures (Bayot and Bragg, 2026). The VITEK-2 Compact automated microbiological identification system and its corresponding cards were utilized, with drug susceptibility results interpreted based on the Clinical and Laboratory Standards Institute (CLSI) 2021 standards (Schuetz et al., 2025). Multidrug resistance (MDR) is defined as resistance to three or more classes of antimicrobial agents (Magiorakos et al., 2012).

### Statistical methods

#### Handling of missing data

Missing data in continuous variables were handled by median imputation. Categorical variables had no missing values. No patients were missing the primary outcome (mortality).

#### Predictor screening and stability selection

We used LASSO regression with 10-fold cross-validation and selected lambda using the 1 − SE rule (λ.1se). We retained predictors with non-zero coefficients as candidates. To evaluate predictor stability, we performed 1,000 bootstrap resampling iterations (each with 10-fold cross-validation) and calculated the selection frequency for each variable. We considered variables with selection frequency >60% as stable prognostic factors.

#### Model development and internal evaluation

Using the final stable predictors, 11 models were developed: logistic regression, random forest, XGBoost, support vector machine (SVM), neural network, elastic net, ridge regression, linear discriminant analysis (LDA), naive Bayes, decision tree, and gradient boosting machine. All models were trained and evaluated using 10-fold cross-validation.

Performance was assessed using discrimination [area under the receiver operating characteristic curve (AUC)], calibration [Brier score; Hosmer–Lemeshow (HL) statistics in the **Supplementary Material**], and threshold-dependent metrics (accuracy, sensitivity, specificity, precision, and F1). Lower Brier scores indicate better probabilistic accuracy (0 = perfect and 0.25 = non-informative given 50% prevalence). Clinical utility was evaluated via decision curve analysis (DCA). Death was the positive class.

#### Bootstrap optimism correction

To correct for overfitting, bootstrap optimism correction was performed (500 iterations, Efron–Gong method) for all models. In each iteration, the model was refit on the bootstrap sample and evaluated on the out-of-bag sample. Optimism (apparent minus test performance) was subtracted from apparent performance to obtain optimism-corrected AUC, calibration intercept, and slope; 95% confidence intervals (CIs) were derived from the distribution of test performance.

#### Survival analysis and risk stratification

For time-to-event analysis, Kaplan–Meier curves were constructed comparing survival distributions across risk groups defined by each model. Time origin was the date of the first positive KP culture. The endpoint was in-hospital death; survivors discharged or transferred were censored on the date of discharge/transfer. Log-rank tests assessed differences between strata.

#### Non-linear relationship exploration

To explore potential non-linearity between continuous predictors and mortality risk, we fitted generalized additive models (GAMs) using restricted maximum likelihood estimation and reported effective degrees of freedom (edf) and p-values.

#### Model interpretability

Model interpretability was explored using SHapley Additive exPlanations (SHAP) analyses. For tree-based models, TreeSHAP was applied; for other models, an approximation method was used.

All analyses were performed using R (version 4.5.2).

## Results

**Figure 1:**
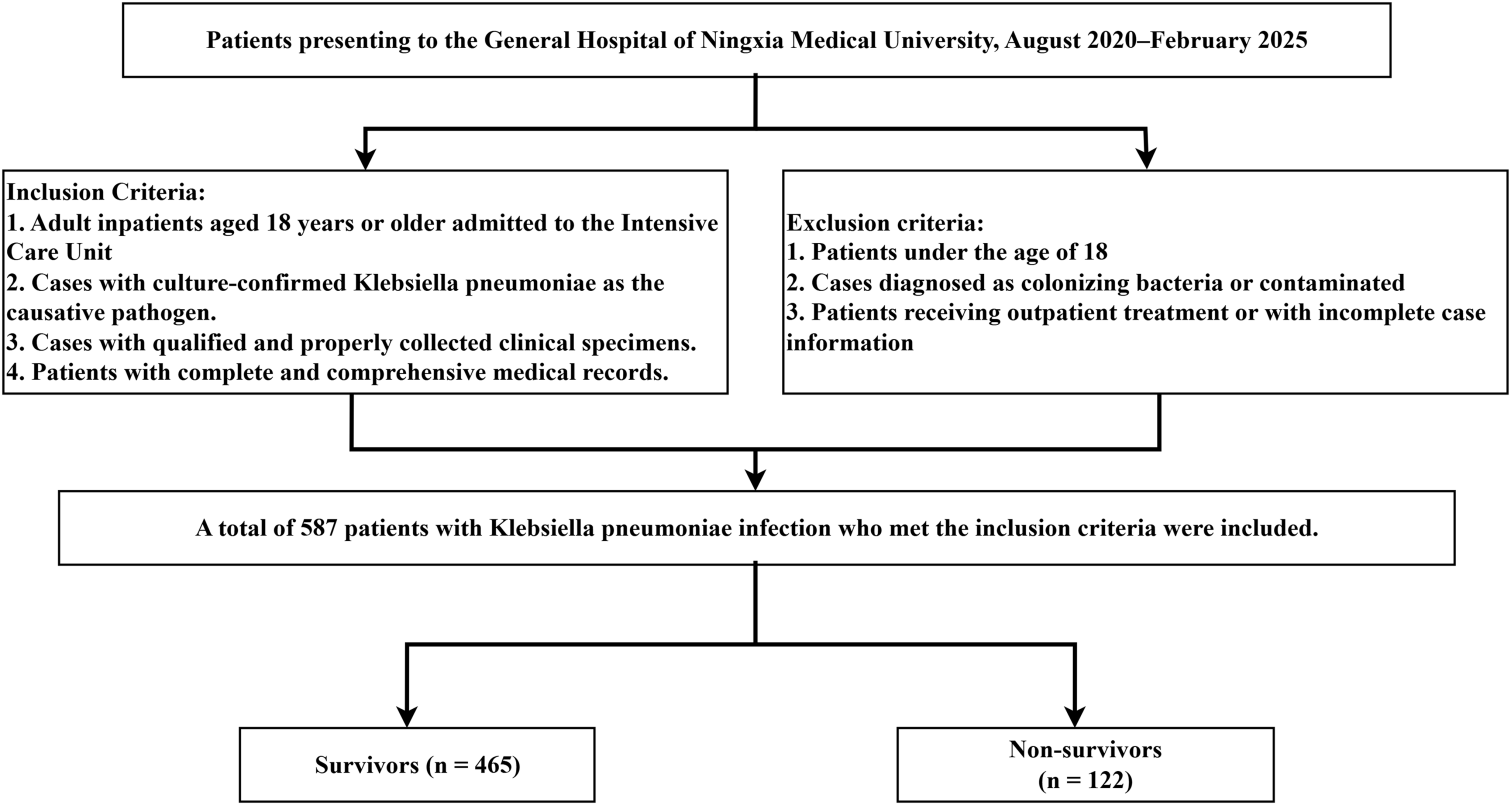
Patient selection flowchart. Adult ICU patients with culture-confirmed KP infection (August 2020–February 2025) were included if aged ≥18 years, with qualified specimens and complete records. Exclusions: age < 18, colonization/contamination, outpatient, and incomplete data. Final cohort: 587 patients (465 survivors, 79.2%; 122 non-survivors, 20.8%). ICU, intensive care unit; KP, *Klebsiella pneumoniae*.

Among 587 ICU patients with culture-confirmed KP infection, 122 (20.8%) died during hospitalization, and 465 (79.2%) survived (**Figure 1**). Baseline characteristics are summarized in **Supplementary Table 1**.

Compared with survivors, non-survivors were older (median 68.0 vs. 58.0 years) and had higher heart rate (median 105.0 vs. 98.0 beats/min) (both p < 0.001). Non-survivors had higher proportions of sepsis, cardiovascular disease, biliary disease, AKI, and hypertension (all p < 0.05), as well as a higher Charlson comorbidity index (median 5.0 vs. 3.0; p < 0.001).

Non-survivors also had higher rates of fungal co-infection, viral co-infection, MDR KP, carbapenem-resistant *K. pneumoniae* (CRKP), and migratory infection (all p < 0.01). In laboratory testing, non-survivors showed lower lymphocyte count, platelet count, and oxygenation index, and higher lactate and multiple organ injury or inflammatory markers (all p < 0.05). They more frequently received vasoactive agents and corticosteroids, had higher APACHE II and SOFA scores, and had lower GCS scores (all p < 0.01).

**Figure 2:**
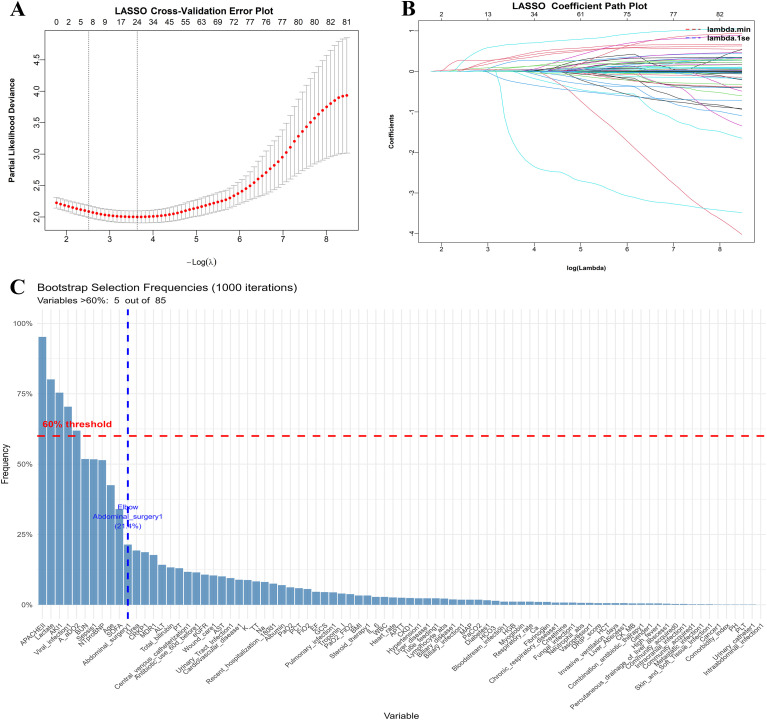
LASSO regression. **(A)** Ten-fold cross-validation error plot showing optimal λ values (λ.min, λ.1se). The top axis indicates the number of non-zero coefficients decreasing from 83 to 1 as regularization increases. **(B)** Coefficient path plot: standardized trajectories of features across log(λ), with vertical lines at λ.min and λ.1se. **(C)** Bootstrap selection frequencies (1,000 iterations) for candidate predictors. Variables with frequency > 60% (dashed line) were selected as stable prognostic predictors. LASSO, least absolute shrinkage and selection operator.

From 85 candidate variables, LASSO regression selected eight potential predictors. After 1,000bootstrap resampling iterations [10-fold cross-validation (CV) each], five variables had selectionfrequency >60% and were identified as stable prognostic predictors (**Supplementary Table 2**, **3**, **Figure 2**): APACHE II, lactate, viral co-infection, AKI, and the A-aDO_2_. Although the lowevents per variable (EPV) raises concerns about stability, the consistently high selectionfrequencies across 1,000 bootstrap iterations (>60% for all five variables, as detailed in **Supplementary Table 3**) suggest that these predictors are relatively robust compared to other candidates. No additional variables exceeded the 60% threshold in any bootstrap run, indicating that the selected set is not driven by random noise alone.

Using these five variables, we built 11 machine learning models. Logistic regression, elasticnet, and ridge regression achieved the highest AUC (0.809), good calibration (HL p > 0.05), Brierscores (0.1286–0.1290), and accuracy (0.821), outperforming other models. Tree-based models showed poorer calibration. **Supplementary Table 4** details the performance metrics. However, the specificity at the conventional 0.5 probability threshold was only 0.336, meaning that more than two-thirds of survivors would be incorrectly flagged as high-risk. This high false-positive rate substantially limits the model’s clinical utility in its current form. To identify a more balanced cutpoint, we calculated Youden’s index based on the logistic regression-predicted probabilities. The optimal threshold was 0.1456, at which sensitivity increased to 0.8115, specificity to 0.6796, positive predictive value to 0.3992, and negative predictive value to 0.9322. This threshold reduces the false-positive rate from 66.4% to 32.0%, although it still misclassifies approximately one-third of survivors.

**Figure 3:**
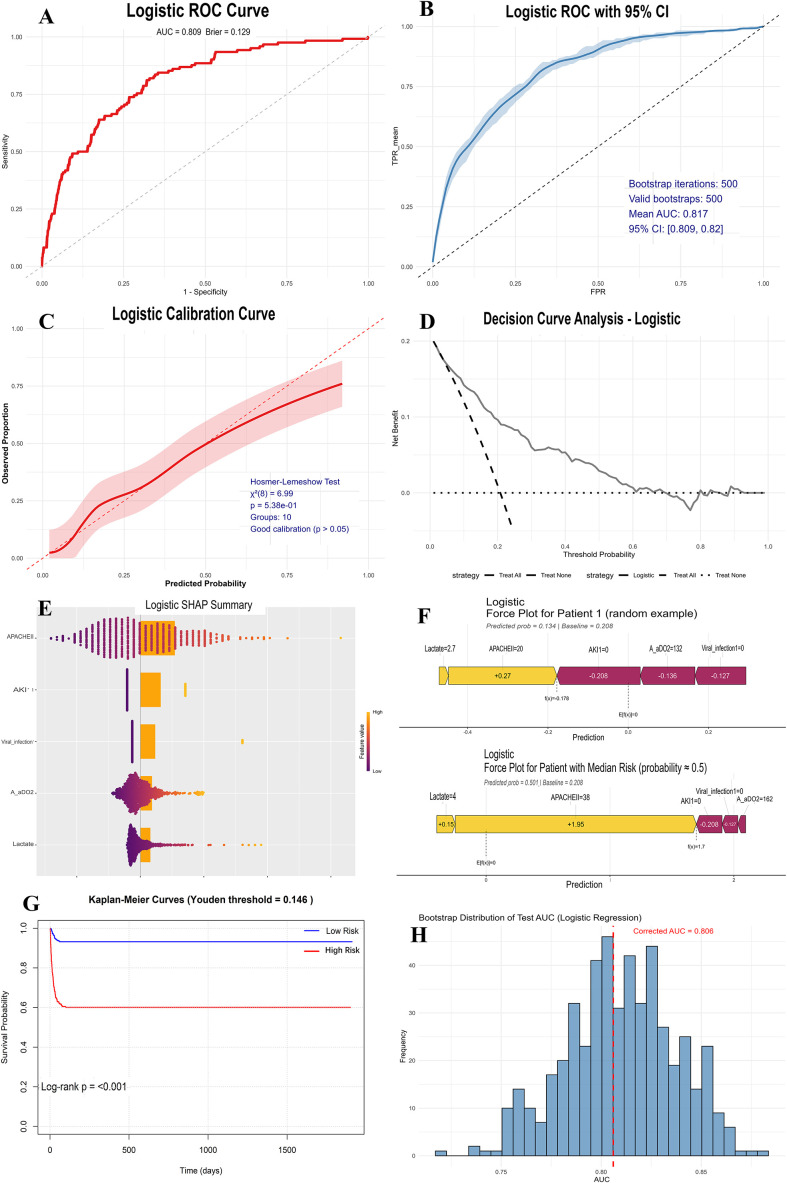
Performance evaluation and clinical validation of the logistic regression model. **(A)** ROC curve: AUC = 0.809, Brier = 0.129. **(B)** Bootstrap-validated ROC (500 iterations): mean AUC = 0.817 (95% CI: 0.809–0.820). **(C)** Calibration curve: HL test χ^2^ = 1.90, df = 8, p = 0.538, indicating good calibration. **(D)** Decision curve: the model provided positive net benefit across threshold probabilities of 0.1–0.6, outperforming “Treat All” and “Treat None” strategies. **(E)** SHAP summary plot: top contributors were APACHE II, AKI, viral infection, A-aDO_2_, and lactate. **(F)** Force plots for a high-risk (predicted probability 0.734) patient and a median-risk (0.5) patient, illustrating individual risk factor contributions. **(G)** Youden-optimized risk score cutoff (total score >48 vs. ≤48). The high-risk group had significantly lower survival than the low-risk group (log-rank p < 0.001). **(H)** Bootstrap distribution of test AUC for the logistic regression model (500 iterations). ROC, receiver operating characteristic; AUC, area under the ROC curve; HL, Hosmer–Lemeshow; APACHE II, Acute Physiology and Chronic Health Evaluation II; AKI, acute kidney injury; A-aDO_2_, alveolar–arterial oxygen gradient.

Decision curve analysis showed that the logistic regression model provided a positive net benefit across a range of threshold probabilities (**Figure 3D**), indicating clinical utility. The net benefit was superior to both the“treat-all” and “treat-none” strategies and better than that of theother machine learning models (**Supplementary Figure 3**).

After 500 bootstrap iterations, the mean AUC was 0.817 (0.809–0.820) for logisticregression, 0.942 (0.919–0.961) for random forest, and 0.921 (0.896–0.946) forXGBoost; the latter two showed instability. Optimism correction for logistic regression yielded corrected AUC = 0.806 (0.755–0.858), calibration intercept = −0.044 (−0.695 to 0.860), and calibration slope = 0.940 (0.597–1.389). The small optimism in AUC (0.014) and near-ideal corrected slope indicate that the model is not severely overfitted (**Supplementary Table 5**; **Figures 3A–C**).

To further quantify the independent association of each predictor with in-hospital mortality inthe final logistic regression model, we performed univariate and multivariate logistic regressionanalyses using the five stable variables. In univariate analysis, all five predictors were significantly associated with mortality (all p < 0.001). In the multivariate model adjusting for all five predictors simultaneously, each predictor remained independently associated with mortality: APACHE II (OR = 1.098, 95% CI: 1.061–1.137, p < 0.001), lactate (OR = 1.102, 95% CI: 1.020–1.198, p = 0.016), viral co-infection (OR = 5.666, 95% CI: 2.790–11.664, p < 0.001), AKI (OR = 2.501, 95% CI: 1.513–4.114, p < 0.001), and the A-aDO_2_ (OR = 1.002, 95% CI: 1.000–1.004, p = 0.018). Detailed results are shown in **Supplementary Table 6**.

**Figure 4:**
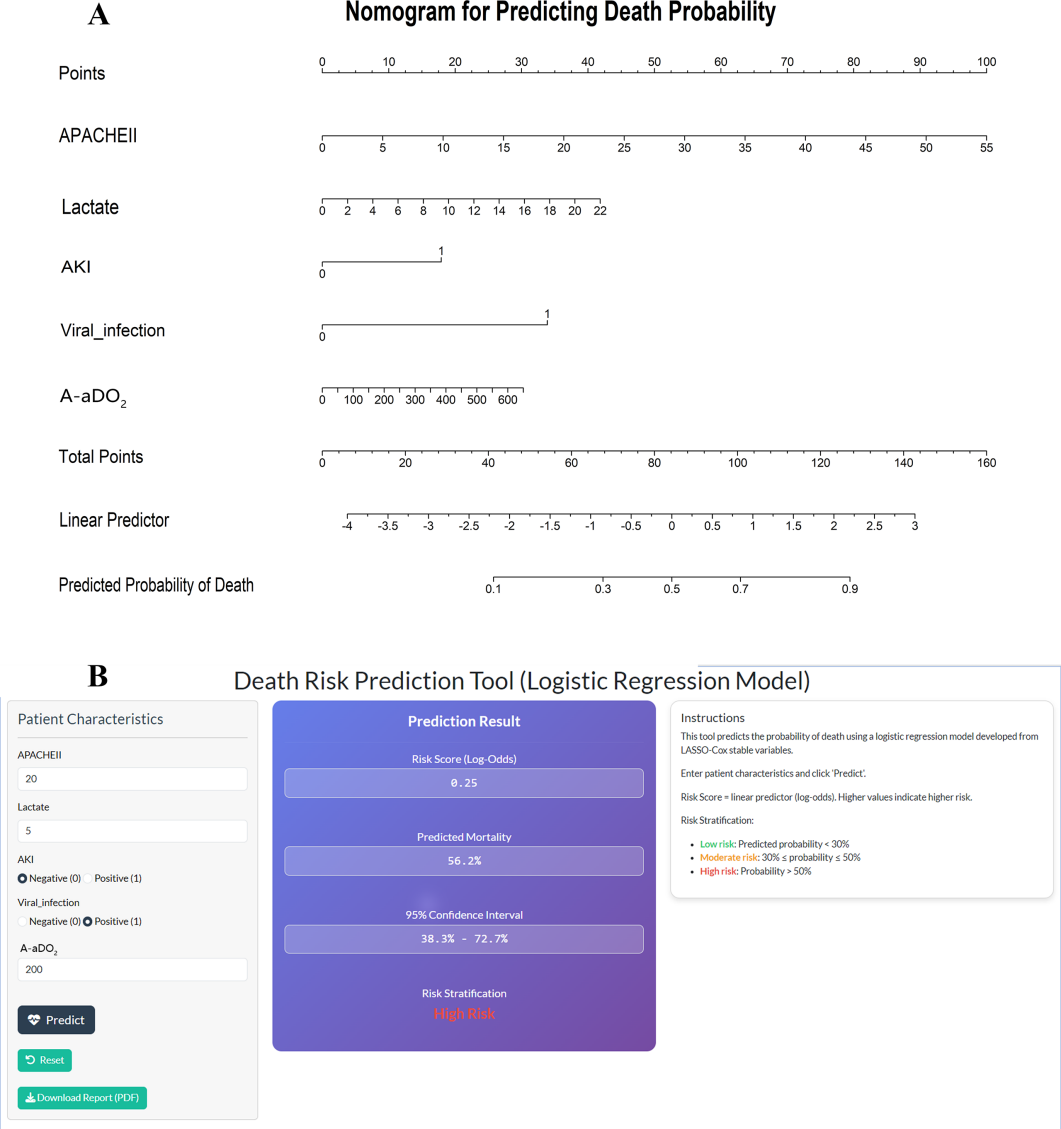
Clinical prediction tools for mortality risk stratification. **(A)** Nomogram based on five LASSO predictors: APACHE II (0–55), lactate (0–22 mmol/L), AKI (0/1), viral infection (0/1), and A-aDO_2_ (0–600 mmHg). Points are assigned to each variable, summed to a total score (0–160), and then converted to predicted mortality probability (0.1–0.9). Higher total points indicate exponentially greater risk. **(B)** Web-based interactive tool (logistic regression model). Example: a patient with APACHE II = 20, lactate = 5 mmol/L, AKI = 0, viral infection = 1, and A-aDO_2_ = 200 mmHg yields a risk score of 0.25, predicted mortality of 56.2% (95% CI: 38.3%–72.7%), and high-risk classification (>50%). The interface includes input fields, automated calculation, and PDF report export for real-time clinical decision support. LASSO, least absolute shrinkage and selection operator; APACHE II, Acute Physiology and Chronic Health Evaluation II; AKI, acute kidney injury; A-aDO_2_, alveolar–arterial oxygen gradient.

Based on the optimal model, logistic regression, a nomogram for predicting mortality rates was constructed along with an interactive web-based scoring system (Prognosis Prediction for Patients with Klebsiella pneumoniae Infection) (**Figure 4**). The total score for each patient was calculated from the nomogram, ranging from 0 to 160 points. Youden’s index was then applied to the logistic regression-predicted probabilities to identify the optimal score cutoff.

Using Youden’s index, an optimal risk score cutoff of 48 points (total score range 0–160) was derived. Patients with a score >48 were classified as high-risk (n = 250), and those ≤48 as low-risk (n = 337). The Kaplan–Meier analysis showed significantly lower survival in the high-risk group (log-rank p < 0.001), as presented in **Figure 3G**. The bootstrap distribution of the test AUC is shown in **Figure 3H**. At this cutoff, the model achieved a sensitivity of 0.8115, a specificity of 0.6796, a positive predictive value of 0.3992, and a negative predictive value of 0.9322. This Youden-optimized binary classification provides a balanced trade-off between sensitivity and specificity.

SHAP analysis demonstrated that in logistic regression (**Figures 3E, F**; **Supplementary Figures 13**–**18**), APACHE II had the highest contribution (SHAP value = 0.54), followed by acute kidney injury (0.32), viral co-infection (0.23), the A-aDO_2_ (0.18), and lactate (0.16). In tree-based models, the A-aDO_2_ ranked second, suggesting that these models more accurately capture its non-linear effects. Dependence plots indicated that APACHE II SHAP values increased sharply above approximately 20 points; the A-aDO_2_ showed an accelerating increase near 165 mmHg; lactate displayed an approximately linear trend. Viral co-infection and acute kidney injury both increased mortality risk. Individual force and waterfall plots illustrated the direction and magnitude of each feature’s contribution, thereby enhancing clinical interpretability.

**Figure 5:**
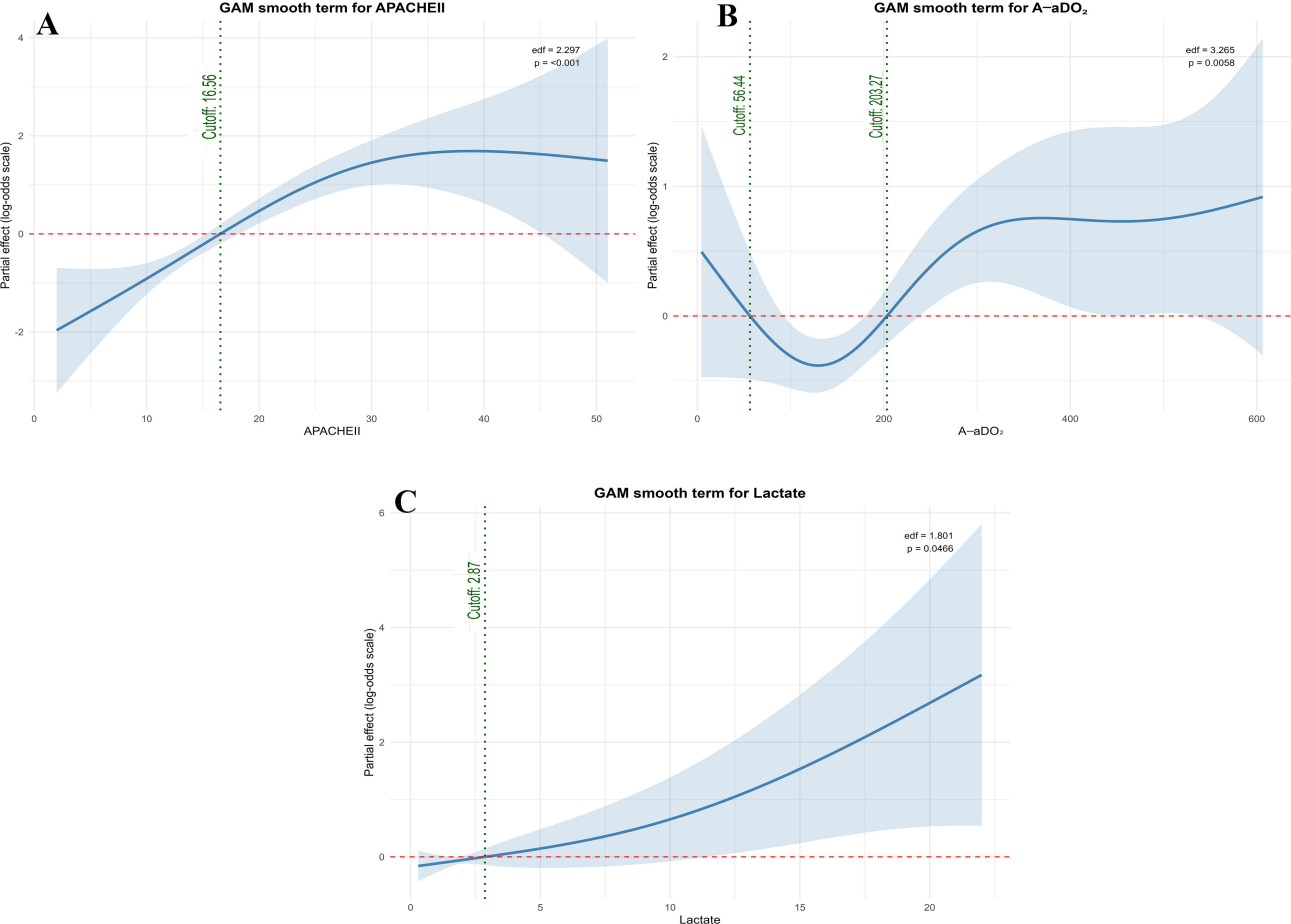
GAM smooth term plots for continuous predictors. **(A)** APACHE II: monotonic near-linear increase (edf = 2.297, p < 0.001); risk rises more steeply above 16.56. **(B)** A-aDO_2_: U-shaped association (edf = 3.265, p = 0.006); critical values at 56.44 and 203.27 mmHg, with an inflection point near 165 mmHg and sharply increased risk above 203.27 mmHg. **(C)** Lactate: convex upward curve (edf = 1.801, p < 0.001), minimal risk below 2.87 mmol/L, and then exponential increase. Shaded areas represent 95% confidence intervals; dashed lines mark clinically relevant cutoffs. These non-linear relationships support flexible modeling for risk prediction in KP infection. GAM, generalized additive model; APACHE II, Acute Physiology and Chronic Health Evaluation II; A-aDO_2_, alveolar–arterial oxygen gradient; KP, *Klebsiella pneumoniae*.

GAM analysis revealed non-linear associations between three variables and mortality risk (**Figure 5**). APACHE II showed significant non-linearity (edf = 2.30, cutoff = 16.56, χ^2^ = 43.68, p < 0.001). Lactate was near-linear (edf = 1.801, cutoff = 2.87 mmol/L). The A-aDO_2_ exhibited the strongest non-linear pattern (edf = 3.27, critical values at 56.44 and 203.27 mmHg, χ^2^ = 12.93, p = 0.006), with an inflection point near 165 mmHg and a sharply increased risk above 203.27 mmHg. Total deviance explained was 19.4% (adjusted R^2^ = 0.194).

## Discussion

In this retrospective study of 587 ICU patients with KP infection, 11 machine learning models were evaluated for predicting mortality, and non-linear relationships of key variables were explored. The main findings were as follows: 1) APACHE II, lactate, viral co-infection, AKI, and the A-aDO_2_ were selected as stable predictors. 2) Logistic regression, elastic net, and ridge regression showed robust discrimination, calibration, and overall performance, while complex tree models overfitted. 3) APACHE II, lactate, and the A-aDO_2_ exhibited non-linear associations with mortality, with a clear threshold for the A-aDO_2_. 4) The logistic regression-based nomogram and web tool enabled individualized risk stratification.

We used LASSO with 10-fold CV for variable selection, followed by 1,000 bootstrap validations to ensure reliability. The final set of stable predictors was directly used for model development. Bootstrap optimism correction revealed only a small optimism in AUC (0.014) and a corrected calibration slope close to 1, confirming that our logistic regression model is not substantially overfitted despite the moderate sample size. The five final risk factors cover host baseline status, organ dysfunction, and pathogen characteristics, reflecting the multifactorial nature of KP prognosis assessment.

APACHE II showed the strongest predictive power (SHAP = 0.54), consistent with previous studies (Pan et al., 2025). GAM analysis revealed a non-linear relationship with mortality risk, with an inflection point at 16.56. Clinically, patients with APACHE II above this threshold may warrant closer monitoring and further assessment rather than relying on linear risk estimates.

Lactate, a marker of tissue hypoperfusion and cellular hypoxia, was significantly associated with mortality risk. GAM analysis showed that risk rose rapidly when lactate exceeded 2.87 mmol/L, consistent with sepsis guideline recommendations on lactate clearance (Lee et al., 2021). This threshold provides a quantitative target for early resuscitation in ICU patients with KP infection.

The A-aDO_2_, an indicator of oxygenation impairment and ventilation–perfusion mismatch, showed the strongest non-linear relationship with mortality risk, with an inflection point near 165 mmHg. Clinically, although the oxygenation index (PaO_2_/FiO_2_) is more commonly used, the A-aDO_2_ may more sensitively reflect gas exchange dysfunction in KP infection, including pulmonary lesions, interstitial injury, and microcirculatory disorders.

Viral co-infection was associated with a significantly higher mortality risk, highlighting its negative prognostic impact. This issue has gained particular attention in ICUs since the COVID-19 pandemic. Routine screening for viral co-infection should be considered in KP-infected patients, especially those with respiratory infections, to enable early identification and timely intervention.

AKI was associated with mortality risk. AKI not only reflects infection severity but also limits antibiotic choices and dosing, worsening prognosis. This finding highlights the need for close renal function monitoring and optimized antibiotic dosing in KP infection treatment.

Among 11 models, traditional regression methods (logistic regression, elastic net, and ridge regression) showed robust discrimination (AUC = 0.809), calibration (HL p > 0.05), and overall performance (Brier score ≈ 0.129), outperforming most complex models. The low Brier score (0.129) of the logistic regression model further supports its excellent calibration, consistent with the calibration curve and the Hosmer–Lemeshow test. Although random forest and XGBoost achieved higher AUCs (0.942 and 0.921, respectively) during bootstrap validation, this suggests overfitting given the modest sample size (n = 587). Regularized regression controls model complexity and enhances generalization. Moreover, logistic regression offers direct coefficient interpretation, aiding clinical understanding, whereas tree models remain less transparent even with SHAP analysis. Thus, simplicity remains valuable in clinical prediction model development.

The logistic regression model achieved a promising AUC of 0.809, but its specificity at the default 0.5 threshold was only 0.336. Using Youden’s index, we identified an alternative cutpoint of 0.1456, which gives a sensitivity of 0.8115 and a specificity of 0.6796. At this threshold, the false-positive rate dropped from 66% to approximately 32%, while the negative predictive value reached 0.9322—meaning that fewer than 7% of patients classified as low-risk would actually die. However, the positive predictive value remained modest (0.3992), indicating that even with the optimized cutpoint, more than half of the patients flagged as high-risk would survive. This trade-off reflects the inherent difficulty of predicting death in a heterogeneous ICU population with a limited sample size. The choice of threshold ultimately depends on the clinical goal: a lower threshold (closer to 0.1456) favors sensitivity and is suitable for screening or triage, whereas a higher threshold (e.g., 0.5) would be preferred when the risks of intervention are high. We recommend that external validation studies prospectively select a threshold based on the intended use and local resources.

GAM analysis revealed non-linear relationships between three key variables and mortality risk, with direct clinical implications. APACHE II had an inflection point at 16.56, aligning with the prior threshold range of 15–20 (Knaus et al., 1985), helping identify high-risk patients for intensive monitoring. Lactate showed a threshold at 2.87 mmol/L, slightly above the traditional sepsis criterion of 2.0 mmol/L but consistent with hyperlactatemia (Singer et al., 2016); even modest lactate elevation signals poor prognosis and warrants early perfusion assessment. The A-aDO_2_ exhibited the most complex non-linearity, with an inflection point near 165 mmHg. Elevated A-aDO_2_ indicates alveolar–capillary gas exchange impairment, seen in KP infection, acute respiratory distress syndrome (ARDS), or pulmonary embolism. Unlike PaO_2_/FiO_2_, the A-aDO_2_ is less influenced by inspired oxygen and more stably reflects lung dysfunction. Thus, incorporating the A-aDO_2_ into respiratory assessment may enable earlier identification of high-risk patients.

The logistic regression-based nomogram and interactive web tool are the main clinical outputs of this study. The nomogram visually presents the five risk factors, allowing bedside calculation of individualized mortality risk. The web tool enables real-time input and risk output, lowering the barrier to use. This binary risk classification (high-risk vs. low-risk) supports not only prognostic assessment but also patient stratification, resource allocation, and clinician–patient communication in clinical trials.

## Limitations

This study has the following limitations.

1. Insufficient sample size for reliable model development.
  The study had only 122 death events but examined 85 candidate predictors, giving an EPV of roughly 1.4, far below the recommended minimum of 10–20. Under these circumstances, even regularized regression with bootstrap stability selection is prone to overfitting and may pick up noise-driven predictors. Consequently, the five variables should be treated as hypothesis-generating rather than definitive, and the reported performance metrics are likely overestimated.
2. Lack of external validation.
  This single-center retrospective study lacked access to an independent dataset for geographic or temporal validation. We considered splitting the cohort by admission date (early period vs. later period), but the total number of deaths (122) was too small to produce a meaningful validation sample; the later period would contain fewer than 50 deaths, making any performance estimate—whether AUC or calibration—too unstable to be trustworthy. Rather than report unreliable numbers, we chose not to perform such a split. Instead, we have developed a concrete plan for prospective external validation: at least three ICUs from different regions of China, targeting 500 patients with a minimum of 100 deaths (to achieve an EPV ≥ 10 for our five predictors). Data collection is estimated to take 18 months, followed by 6 months for analysis. Until this validation is completed, the model should not be used in clinical practice.
3. Treatment process variables not captured.
  The model did not include treatment process variables (such as types of antibiotics used, timing of administration, and whether pharmacokinetic/pharmacodynamic targets were achieved), which similarly affect prognosis in actual clinical settings.
4. Dynamic treatment-related factors not incorporated.
  Dynamic treatment-related variables (such as antibiotic adjustments and fluid resuscitation volumes) were not incorporated, indicating that there may be further room for improvement in the predictive capability of this model.
5. Detection bias for viral co-infection and lack of virus-type stratification.
  Viral testing was not protocol-mandated; it was ordered based on clinical judgment. Consequently, patients with more severe illness or respiratory symptoms were more likely to be tested, which may have inflated the observed association between viral co-infection and mortality. In addition, we attempted to stratify by individual virus types (e.g., SARS-CoV-2, CMV, and influenza), but the number of positive cases for each virus was too small to yield meaningful estimates. Future studies with systematic viral screening and larger sample sizes are needed to disentangle the prognostic role of specific viruses.

## Future directions

Based on the results of this study, further exploration can be conducted in the following areas: 1) conduct multicenter prospective studies to validate the generalizability of the model, 2) incorporate treatment process variables to construct dynamic predictive models, 3) investigate the immune mechanisms of virus–bacteria co-infection to provide a basis for targeted therapy, and 4) implement interventional studies to evaluate whether intensified treatment based on model stratification can improve patient prognosis.

## Conclusion

In summary, five predictors (APACHE II, lactate, viral co-infection, AKI, and the A-aDO_2_) were identified as associated with mortality in ICU patients with culture-confirmed *K. pneumoniae* infection. Among the 11 models tested, logistic regression offered the best balance of discrimination and calibration, and non-linear patterns were observed for three continuous variables. Given the limited number of outcome events relative to the number of candidate predictors, these findings should be considered hypothesis-generating. The proposed model is not ready for clinical use and requires external validation in a larger, independent cohort.

## Data Availability

The raw data supporting the conclusions of this article will be made available by the authors, without undue reservation.
