# Supplementary material for: Machine learning-assisted prognostic model for mortality in ICU patients with culture-confirmed Klebsiella pneumoniae infection: a preliminary single-center retrospective cohort study
Source: Front Cell Infect Microbiol. 2026 Jun 19;16:1858732. doi: 10.3389/fcimb.2026.1858732 (PMC13328191; doi:10.3389/fcimb.2026.1858732)
Supplement: Supplementary file 1 [file Table1.docx]

**Supplementary Tables S1–S6**

**Supplementary Table S1. Baseline Characteristics of ICU Patients With Culture-Confirmed Klebsiella pneumoniae Infection, Stratified by In-Hospital Mortality**

Demographics, clinical characteristics, comorbidities, infection features, laboratory data, treatments, and severity scores of survivors and non‑survivors.

*Abbreviations:*

AKI, acute kidney injury; APACHE II, Acute Physiology and Chronic Health Evaluation II; APTT, activated partial thromboplastin time; BMI, body mass index; CKD, chronic kidney disease; CK‑MB, creatine kinase myocardial band; CRKP, carbapenem‑resistant Klebsiella pneumoniae; GCS, Glasgow Coma Scale; HCT, hematocrit; HGB, hemoglobin; IL‑6, interleukin‑6; MAP, mean arterial pressure; MDR, multidrug‑resistant; PaO₂/FiO₂, ratio of arterial oxygen partial pressure to fractional inspired oxygen; A‑aDO₂, alveolar‑arterial oxygen gradient; PCT, procalcitonin; PH, potential of hydrogen; PLT, platelet count; PT, prothrombin time; SOFA, Sequential Organ Failure Assessment; TT, thrombin time; WBC, white blood cell count.

*Footnotes:*

Data are presented as median (interquartile range) for continuous variables and number (percentage) for categorical variables. p-values were calculated using Mann-Whitney U test for continuous variables and Chi-square test or Fisher's exact test for categorical variables, as appropriate.

| **Characteristic** | **Survivors (n = 465)** | **Non-survivors (n = 122)** | **P value** |
| --- | --- | --- | --- |
| **Demographics** | | | |
| Age, years | 58.00 (48.00-68.00) | 68.00 (56.25-76.00) | <0.001 |
| Sex |  |  | 0.349 |
| Female | 113 (24.3%) | 35 (28.7%) |  |
| Male | 352 (75.7%) | 87 (71.3%) |  |
| **Clinical characteristics** | | | |
| Community-acquired infection |  |  | 0.760 |
| No | 212 (45.6%) | 58 (47.5%) |  |
| Yes | 253 (54.4%) | 64 (52.5%) |  |
| Body mass index, kg/m² | 23.88 (22.04-25.51) | 24.09 (22.04-25.39) | 0.748 |
| Mean arterial pressure, mmHg | 86.33 (77.33-96.33) | 82.50 (74.67-94.75) | 0.081 |
| High fever (>39°C) |  |  | 0.020 |
| No | 303 (65.2%) | 65 (53.3%) |  |
| Yes | 162 (34.8%) | 57 (46.7%) |  |
| Heart rate, beats/min | 98.00 (84.00-117.00) | 105.00 (90.50-126.75) | <0.001 |
| Respiratory rate, breaths/min | 20.00 (20.00-20.00) | 20.00 (20.00-20.00) | 0.119 |
| **Comorbidities** | | | |
| Malignancy |  |  | 0.408 |
| No | 347 (74.6%) | 96 (78.7%) |  |
| Yes | 118 (25.4%) | 26 (21.3%) |  |
| Sepsis |  |  | <0.001 |
| No | 279 (60.0%) | 30 (24.6%) |  |
| Yes | 186 (40.0%) | 92 (75.4%) |  |
| Chronic respiratory disease |  |  | 0.565 |
| No | 399 (85.8%) | 102 (83.6%) |  |
| Yes | 66 (14.2%) | 20 (16.4%) |  |
| Cardiovascular disease |  |  | <0.001 |
| No | 378 (81.3%) | 80 (65.6%) |  |
| Yes | 87 (18.7%) | 42 (34.4%) |  |
| Liver disease |  |  | 0.346 |
| No | 355 (76.3%) | 88 (72.1%) |  |
| Yes | 110 (23.7%) | 34 (27.9%) |  |
| Biliary disease |  |  | 0.004 |
| No | 397 (85.4%) | 90 (73.8%) |  |
| Yes | 68 (14.6%) | 32 (26.2%) |  |
| Chronic kidney disease |  |  | 0.169 |
| No | 451 (97.0%) | 115 (94.3%) |  |
| Yes | 14 (3.0%) | 7 (5.7%) |  |
| Acute kidney injury |  |  | <0.001 |
| No | 391 (84.1%) | 63 (51.6%) |  |
| Yes | 74 (15.9%) | 59 (48.4%) |  |
| Hypertension |  |  | 0.016 |
| No | 330 (71.0%) | 72 (59.0%) |  |
| Yes | 135 (29.0%) | 50 (41.0%) |  |
| Diabetes mellitus |  |  | 0.097 |
| No | 331 (71.2%) | 77 (63.1%) |  |
| Yes | 134 (28.8%) | 45 (36.9%) |  |
| Charlson comorbidity index | 3.00 (1.00-6.00) | 5.00 (3.00-6.00) | <0.001 |
| **Recent history** | | | |
| Hospitalization within previous 180 days |  |  | <0.001 |
| No | 369 (79.4%) | 71 (58.2%) |  |
| Yes | 96 (20.6%) | 51 (41.8%) |  |
| Antibiotic use within previous 60 days |  |  | <0.001 |
| No | 370 (79.6%) | 71 (58.2%) |  |
| Yes | 95 (20.4%) | 51 (41.8%) |  |
| **Infection characteristics** | | | |
| Fungal co-infection |  |  | 0.002 |
| No | 307 (66.0%) | 62 (50.8%) |  |
| Yes | 158 (34.0%) | 60 (49.2%) |  |
| Viral co-infection |  |  | <0.001 |
| No | 446 (95.9%) | 98 (80.3%) |  |
| Yes | 19 (4.1%) | 24 (19.7%) |  |
| Multidrug-resistant K. pneumoniae |  |  | <0.001 |
| No | 370 (79.6%) | 78 (63.9%) |  |
| Yes | 95 (20.4%) | 44 (36.1%) |  |
| Carbapenem-resistant K. pneumoniae |  |  | <0.001 |
| No | 425 (91.4%) | 98 (80.3%) |  |
| Yes | 40 (8.6%) | 24 (19.7%) |  |
| Metastatic infection |  |  | 0.001 |
| No | 311 (66.9%) | 62 (50.8%) |  |
| Yes | 154 (33.1%) | 60 (49.2%) |  |
| Pulmonary infection |  |  | <0.001 |
| No | 126 (27.1%) | 14 (11.5%) |  |
| Yes | 339 (72.9%) | 108 (88.5%) |  |
| Intra-abdominal infection |  |  | 0.049 |
| No | 287 (61.7%) | 63 (51.6%) |  |
| Yes | 178 (38.3%) | 59 (48.4%) |  |
| Biliary tract infection |  |  | 0.005 |
| No | 424 (91.2%) | 100 (82.0%) |  |
| Yes | 41 (8.8%) | 22 (18.0%) |  |
| Urinary tract infection |  |  | 0.011 |
| No | 450 (96.8%) | 111 (91.0%) |  |
| Yes | 15 (3.2%) | 11 (9.0%) |  |
| Skin and soft tissue infection |  |  | 0.838 |
| No | 434 (93.3%) | 115 (94.3%) |  |
| Yes | 31 (6.7%) | 7 (5.7%) |  |
| Bloodstream infection |  |  | <0.001 |
| No | 400 (86.0%) | 87 (71.3%) |  |
| Yes | 65 (14.0%) | 35 (28.7%) |  |
| **Laboratory findings: complete blood count** | | | |
| White blood cell count, ×10⁹/L | 10.13 (7.07-14.39) | 10.99 (5.81-15.15) | 0.523 |
| Absolute neutrophil count, ×10⁹/L | 8.36 (5.56-12.03) | 9.79 (4.34-12.27) | 0.706 |
| Absolute lymphocyte count, ×10⁹/L | 0.83 (0.53-1.30) | 0.62 (0.37-1.10) | <0.001 |
| Hemoglobin | 106.00 (90.00-131.00) | 100.00 (88.00-124.00) | 0.223 |
| Hematocrit, % | 32.80 (27.50-39.20) | 30.40 (26.92-37.88) | 0.149 |
| Platelet count, ×10⁹/L | 155.00 (93.00-221.00) | 134.00 (77.25-181.75) | 0.011 |
| **Laboratory findings: blood gas analysis** | | | |
| pH | 7.37 (7.30-7.42) | 7.38 (7.29-7.43) | 0.638 |
| PaO₂/FiO₂ ratio | 220.50 (165.71-290.00) | 167.70 (113.54-241.17) | <0.001 |
| PaCO₂ | 38.30 (33.00-44.40) | 37.65 (32.32-46.98) | 0.899 |
| PaO₂ | 92.30 (73.40-118.00) | 81.45 (67.35-104.50) | <0.001 |
| FiO₂ | 45.00 (40.00-50.00) | 50.00 (40.00-60.00) | <0.001 |
| A–aDO₂ | 160.10 (120.70-215.72) | 214.64 (146.71-294.56) | <0.001 |
| Sodium | 141.00 (138.00-145.00) | 140.00 (136.67-146.00) | 0.345 |
| Potassium | 3.80 (3.40-4.10) | 3.90 (3.50-4.38) | 0.054 |
| Calcium | 1.14 (1.09-1.19) | 1.15 (1.09-1.22) | 0.485 |
| Bicarbonate | 21.80 (19.10-25.10) | 22.50 (18.12-26.20) | 0.831 |
| **Laboratory findings: metabolic panel** | | | |
| Lactate, mmol/L | 1.50 (1.00-2.50) | 2.13 (1.30-4.47) | <0.001 |
| Urea | 6.57 (4.75-9.13) | 10.29 (6.98-15.10) | <0.001 |
| Blood urea nitrogen | 3.29 (2.37-4.57) | 5.14 (3.49-7.55) | <0.001 |
| Estimated glomerular filtration rate | 99.55 (75.24-112.72) | 67.29 (34.32-97.56) | <0.001 |
| Creatinine | 69.70 (54.00-95.90) | 92.70 (63.60-153.48) | <0.001 |
| **Laboratory findings: liver function** | | | |
| Total bilirubin | 19.70 (12.90-35.10) | 24.82 (14.18-56.68) | 0.012 |
| Albumin | 27.81 (24.10-32.00) | 27.70 (24.33-31.35) | 0.802 |
| Aspartate aminotransferase | 42.80 (27.20-89.20) | 53.45 (30.93-124.10) | 0.036 |
| Alanine aminotransferase | 38.20 (23.50-74.50) | 41.45 (24.93-75.98) | 0.305 |
| **Laboratory findings: cardiac biomarkers** | | | |
| Cardiac troponin I, ng/mL | 0.02 (0.01-0.10) | 0.05 (0.01-0.20) | <0.001 |
| Myoglobin, ng/mL | 250.70 (107.20-600.00) | 304.64 (128.47-909.22) | 0.101 |
| CK-MB, U/L | 2.36 (0.84-7.34) | 2.50 (1.04-6.31) | 0.546 |
| NT-proBNP | 169 (36.3%); 296 (63.7%) | 10 (8.2%); 112 (91.8%) | <0.001 |
| **Laboratory findings: coagulation profile** | | | |
| Prothrombin time, s | 13.10 (11.90-14.90) | 14.55 (12.33-17.20) | <0.001 |
| Activated partial thromboplastin time, s | 30.80 (28.10-34.50) | 32.25 (29.02-39.75) | 0.002 |
| Fibrinogen, g/L | 3.47 (2.56-4.61) | 3.43 (2.46-4.80) | 0.993 |
| Thrombin time, s | 14.60 (13.30-16.10) | 15.80 (14.00-18.25) | <0.001 |
| **Laboratory findings: inflammatory markers** | | | |
| Procalcitonin, ng/mL | 1.30 (0.22-12.00) | 4.05 (1.00-14.50) | <0.001 |
| Interleukin-6, pg/mL | 359.35 (86.70-600.00) | 388.50 (182.25-708.75) | 0.015 |
| **Treatments and procedures** | | | |
| Duration of invasive mechanical ventilation | 78.04 (14.47-235.42) | 119.13 (19.00-301.50) | 0.033 |
| Vasopressor use |  |  | <0.001 |
| No | 104 (22.4%) | 11 (9.0%) |  |
| Yes | 361 (77.6%) | 111 (91.0%) |  |
| Corticosteroid therapy |  |  | 0.018 |
| No | 167 (35.9%) | 30 (24.6%) |  |
| Yes | 298 (64.1%) | 92 (75.4%) |  |
| Urinary catheterization |  |  | 0.391 |
| No | 17 (3.7%) | 2 (1.6%) |  |
| Yes | 448 (96.3%) | 120 (98.4%) |  |
| Tube feeding |  |  | 0.684 |
| No | 245 (52.7%) | 67 (54.9%) |  |
| Yes | 220 (47.3%) | 55 (45.1%) |  |
| History of abdominal surgery |  |  | 0.033 |
| No | 223 (48.0%) | 72 (59.0%) |  |
| Yes | 242 (52.0%) | 50 (41.0%) |  |
| Wound care |  |  | 0.208 |
| No | 0 (0.0%) | 1 (0.8%) |  |
| Yes | 465 (100.0%) | 121 (99.2%) |  |
| Central venous catheterization |  |  | 0.208 |
| No | 0 (0.0%) | 1 (0.8%) |  |
| Yes | 465 (100.0%) | 121 (99.2%) |  |
| Percutaneous drainage of liver abscess |  |  | 0.242 |
| No | 421 (90.5%) | 106 (86.9%) |  |
| Yes | 44 (9.5%) | 16 (13.1%) |  |
| **Severity scores** | | | |
| APACHE II score | 15.00 (11.00-20.00) | 22.00 (18.00-28.00) | <0.001 |
| SOFA score | 8.00 (6.00-12.00) | 12.00 (9.00-14.75) | <0.001 |
| Glasgow Coma Scale score | 15.00 (12.00-15.00) | 12.00 (9.00-15.00) | <0.001 |
| **Other variables** | | | |
| Combination antibiotic therapy |  |  | 0.001 |
| No | 269 (57.8%) | 50 (41.0%) |  |
| Yes | 196 (42.2%) | 72 (59.0%) |  |
| Liver abscess |  |  | 0.242 |
| No | 421 (90.5%) | 106 (86.9%) |  |
| Yes | 44 (9.5%) | 16 (13.1%) |  |

**Supplementary Table S2. Variables Selected by Least Absolute Shrinkage and Selection Operator (LASSO) Regression**

From 85 candidate predictors (587 patients, 122 deaths), LASSO with 10‑fold CV identified 8 prognostic predictors at lambda.min = 0.0264 and lambda.1se = 0.0807. Selected variables with coefficients and odds ratios: viral infection (coef 0.298, OR 1.347), AKI (coef 0.275, OR 1.316), sepsis (coef 0.066, OR 1.068), lactate (coef 0.054, OR 1.056), NT‑proBNP (coef 0.054, OR 1.055), APACHE II (coef 0.042, OR 1.042), BUN (coef 0.006, OR 1.006), and A‑aDO₂ (coef 0.001, OR 1.001).

*Abbreviations:*

AKI, acute kidney injury; APACHE II, Acute Physiology and Chronic Health Evaluation II; BUN, blood urea nitrogen; OR, odds ratio; NT‑proBNP, N‑terminal pro‑brain natriuretic peptide; A‑aDO₂, alveolar‑arterial oxygen gradient.

*Footnotes:*

Variables with non‑zero coefficients at the optimal lambda are listed. ORs are exponentiated coefficients from LASSO. All eight were retained as predictors.

| **Predictor** | **Coefficient (β)** | **exp(β)** |
| --- | --- | --- |
| Viral co-infection | 0.297793923 | 1.346884 |
| Acute kidney injury | 0.274972189 | 1.316494 |
| Sepsis | 0.065715707 | 1.067923 |
| Lactate | 0.054167142 | 1.055661 |
| NT-proBNP | 0.053629003 | 1.055093 |
| APACHE II score | 0.041586066 | 1.042463 |
| Blood urea nitrogen | 0.005694790 | 1.005711 |
| A–aDO₂ | 0.000566237 | 1.000566 |

**Supplementary Table S3. Bootstrap Stability Selection Results for Candidate Predictors**

Among 85 candidate predictors, bootstrap resampling (1000 iterations) identified variables with selection frequency >0.1. Five highly stable variables (frequency >60%): APACHE II (0.952), lactate (0.801), AKI (0.754), viral infection (0.704), and A‑aDO₂ (0.619). Moderately stable (0.4–0.6): BUN (0.518), sepsis (0.517), NT‑proBNP (0.514), age (0.425), and SOFA (0.340). Lower frequency selections (0.1–0.3): abdominal surgery (0.214), urea (0.193), CRKP (0.187), MDR (0.177), ALT (0.142), total bilirubin (0.133), PT (0.130), central venous catheterization (0.117), antibiotic use within 60 days (0.115), eGFR (0.107), wound care (0.104), and AST (0.101).

*Abbreviations:*

AKI, acute kidney injury; APACHE II, Acute Physiology and Chronic Health Evaluation II; ALT, alanine aminotransferase; AST, aspartate aminotransferase; BUN, blood urea nitrogen; CRKP, carbapenem‑resistant Klebsiella pneumoniae; eGFR, estimated glomerular filtration rate; MDR, multidrug‑resistant; NT‑proBNP, N‑terminal pro‑brain natriuretic peptide; PT, prothrombin time; SOFA, Sequential Organ Failure Assessment; A‑aDO₂, alveolar‑arterial oxygen gradient.

*Footnote:*

Selection frequency is the proportion of bootstrap iterations in which LASSO selected the variable. Frequency >0.60 defines highly stable predictors.

| **Predictor** | **Selection frequency** |
| --- | --- |
| APACHE II score | 0.952 |
| Lactate | 0.801 |
| Acute kidney injury | 0.754 |
| Viral co-infection | 0.704 |
| A–aDO₂ | 0.619 |
| Blood urea nitrogen | 0.518 |
| Sepsis | 0.517 |
| NT-proBNP | 0.514 |
| Age | 0.425 |
| SOFA score | 0.340 |
| History of abdominal surgery | 0.214 |
| Urea | 0.193 |
| Carbapenem-resistant K. pneumoniae | 0.187 |
| Multidrug-resistant K. pneumoniae | 0.177 |
| Alanine aminotransferase | 0.142 |
| Total bilirubin | 0.133 |
| Prothrombin time | 0.130 |
| Central venous catheterization | 0.117 |
| Antibiotic use within previous 60 days | 0.115 |
| Estimated glomerular filtration rate | 0.107 |
| Wound care | 0.104 |
| Aspartate aminotransferase | 0.101 |

**Supplementary Table S4. Predictive Performance of 11 Models for In-Hospital Mortality**

Performance metrics of 11 machine learning models built on 5 stable risk factors from bootstrap validation. Models were evaluated via 10-fold cross-validation on AUC, Brier score, accuracy, sensitivity, specificity, precision, F1 score, and Hosmer-Lemeshow (HL) test.

Logistic regression performed best overall: AUC 0.809, Brier 0.1290, accuracy 0.821, sensitivity 0.948, specificity 0.336, precision 0.845, F1 0.894, and good calibration (HL P = 0.538). Elastic net (AUC 0.809, Brier 0.1288, HL P = 0.492) and ridge regression (AUC 0.809, Brier 0.1286, HL P = 0.539) showed nearly identical performance with good calibration. LDA (AUC 0.807, Brier 0.1325, HL P = 0.007), Naive Bayes (AUC 0.805, Brier 0.1709, HL P < 0.001), random forest (AUC 0.785, Brier 0.1311, HL P < 0.001), decision tree (AUC 0.751, Brier 0.1451, HL P < 0.001), XGBoost (AUC 0.733, Brier 0.1606, HL P < 0.001), and SVM (AUC 0.633, Brier 0.1442, HL P < 0.001) exhibited poor calibration. Neural network (AUC 0.714, Brier 0.1705) and gradient boosting (AUC 0.500, Brier 0.2078, sensitivity 1.000, specificity 0.000) had HL not applicable.

*Abbreviations:*

AUC, area under the ROC curve; Brier, Brier score; F1, F1 score; HL, Hosmer‑Lemeshow test; LDA, linear discriminant analysis; SVM, support vector machine; XGBoost, extreme gradient boosting.

*Footnotes:*

Metrics are averages from 10‑fold CV. HL P > 0.05 = good calibration; < 0.05 = poor calibration. HL not applicable for neural network and gradient boosting. Lower Brier indicates better calibration. Brier score ranges from 0 (perfect prediction) to 0.25 (non‑informative prediction for a binary outcome with 50% prevalence). Lower values indicate better overall predictive accuracy.

| **Model** | **AUC** | **Brier score** | **Accuracy** | **Sensitivity** | **Specificity** | **Precision** | **F1 score** | **HL χ²** | **df** | **P value** | **Calibration** |
| --- | --- | --- | --- | --- | --- | --- | --- | --- | --- | --- | --- |
| Logistic regression | 0.809 | 0.1290 | 0.821 | 0.948 | 0.336 | 0.845 | 0.894 | 6.992 | 8 | 5.38e-01 | Good calibration |
| Elastic net | 0.809 | 0.1288 | 0.821 | 0.951 | 0.328 | 0.844 | 0.894 | 7.418 | 8 | 4.92e-01 | Good calibration |
| Ridge regression | 0.809 | 0.1286 | 0.821 | 0.953 | 0.320 | 0.842 | 0.894 | 6.977 | 8 | 5.39e-01 | Good calibration |
| Linear discriminant analysis | 0.807 | 0.1325 | 0.823 | 0.933 | 0.402 | 0.856 | 0.893 | 21.032 | 8 | 7.06e-03 | Poor calibration |
| Naive Bayes | 0.805 | 0.1709 | 0.799 | 0.882 | 0.484 | 0.867 | 0.874 | 6,947.971 | 8 | 0.00e+00 | Poor calibration |
| Random forest | 0.785 | 0.1311 | 0.819 | 0.948 | 0.328 | 0.843 | 0.893 | 140.133 | 8 | 0.00e+00 | Poor calibration |
| Decision tree | 0.751 | 0.1451 | 0.819 | 0.927 | 0.410 | 0.857 | 0.890 | 42.683 | 8 | 1.01e-06 | Poor calibration |
| XGBoost | 0.733 | 0.1606 | 0.790 | 0.897 | 0.385 | 0.848 | 0.871 | 980.128 | 8 | 0.00e+00 | Poor calibration |
| Neural network | 0.714 | 0.1705 | 0.787 | 0.903 | 0.344 | 0.840 | 0.870 |  |  |  |  |
| Support vector machine | 0.633 | 0.1442 | 0.813 | 0.955 | 0.270 | 0.833 | 0.890 | 38.931 | 8 | 5.06e-06 | Poor calibration |
| Gradient boosting machine | 0.500 | 0.2078 | 0.792 | 1.000 | 0.000 | 0.792 | 0.884 |  |  |  |  |

**Supplementary Table S5. Bootstrap Optimism-Corrected Performance of the Final Logistic Regression Model**

Apparent (original) performance, optimism estimates, and optimism‑corrected performance with 95% confidence intervals for the final logistic regression model, including area under the receiver operating characteristic curve (AUC), calibration intercept, and calibration slope.

*Abbreviations:*

AUC, area under the receiver operating characteristic curve; CI, confidence interval.

*Footnotes:*

Apparent performance was calculated on the full dataset. Optimism was estimated as the mean difference between apparent and test performance across 500 bootstrap iterations (Efron‑Gong method).

| **Metric** | **Apparent performance** | **Optimism estimate** | **Optimism-corrected performance (95% CI)** |
| --- | --- | --- | --- |
| AUC | 0.820 | 0.014 | 0.806 (0.755–0.858) |
| Calibration intercept | 3.25e-14 | 0.044 | −0.044 (−0.695 to 0.860) |
| Calibration slope | 1.000 | 0.060 | 0.940 (0.597–1.389) |

**Supplementary Table S6. Univariate and Multivariate Logistic Regression Analysis of Predictors for Mortality Model**

Univariate (each variable alone) and multivariate (all five variables together) logistic regression results for the five stable predictors (APACHE II, lactate, viral co‑infection, acute kidney injury, and alveolar‑arterial oxygen gradient) identified by LASSO and bootstrap stability selection. Odds ratios (OR) with 95% confidence intervals (CI) and P values are shown.

*Abbreviations:*

AKI, acute kidney injury; APACHE II, Acute Physiology and Chronic Health Evaluation II; CI, confidence interval; OR, odds ratio; A‑aDO₂, alveolar‑arterial oxygen gradient.

*Footnotes:*

| **Variable** | **Univariate OR (95% CI)** | **P value** | **Multivariate OR (95% CI)** | | **P value** |
| --- | --- | --- | --- | --- | --- |
| **APACHE II** | 1.138 (1.104–1.175) | <0.001 | 1.098 (1.061–1.137) | <0.001 | |
| **Lactate** | 1.234 (1.145–1.342) | <0.001 | 1.102 (1.020–1.198) | 0.016 | |
| **Viral co‑infection** | 5.749 (3.039–11.023) | <0.001 | 5.666 (2.790–11.664) | <0.001 | |
| **AKI** | 4.948 (3.212–7.652) | <0.001 | 2.501 (1.513–4.114) | <0.001 | |
| **A‑aDO₂** | 1.005 (1.003–1.007) | <0.001 | 1.002 (1.000–1.004) | 0.018 | |

ORs were calculated using logistic regression with in‑hospital death as the outcome (positive class = death). Univariate models included each predictor separately. Multivariate model included all five predictors simultaneously. P values < 0.05 were considered statistically significant. Missing data were handled by median imputation for continuous variables and mode imputation for categorical variables (no missing values in the final analysis after imputation).
