# Supplementary material for: Machine learning-assisted prognostic model for mortality in ICU patients with culture-confirmed Klebsiella pneumoniae infection: a preliminary single-center retrospective cohort study
Source: Front Cell Infect Microbiol. 2026 Jun 19;16:1858732. doi: 10.3389/fcimb.2026.1858732 (PMC13328191; doi:10.3389/fcimb.2026.1858732)
Supplement: Supplementary file 2 [file Table2.docx]

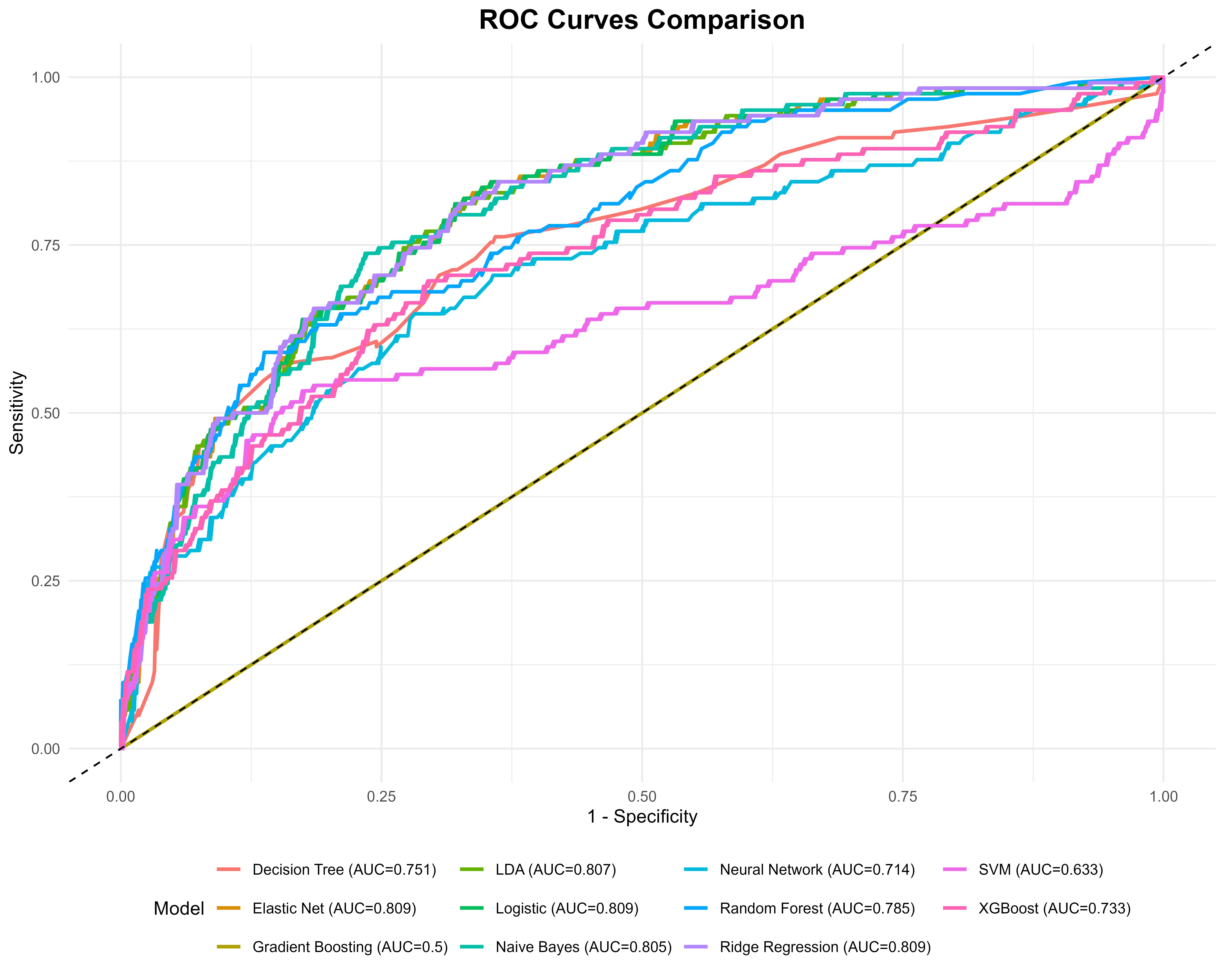


**Supplement Figure S1. ROC curves of 11 machine learning models.**

Logistic regression, elastic net, and ridge regression achieved the highest AUC (all 0.809), followed by LDA (0.807), Naive Bayes (0.805), random forest (0.785), decision tree (0.751), XGBoost (0.733), neural network (0.714), and SVM (0.633). Gradient boosting had AUC 0.5. The top curves cluster in the upper‑left region, indicating that regularized linear models performed as well as or better than complex algorithms, supporting the choice of logistic regression for its simplicity and clinical utility.


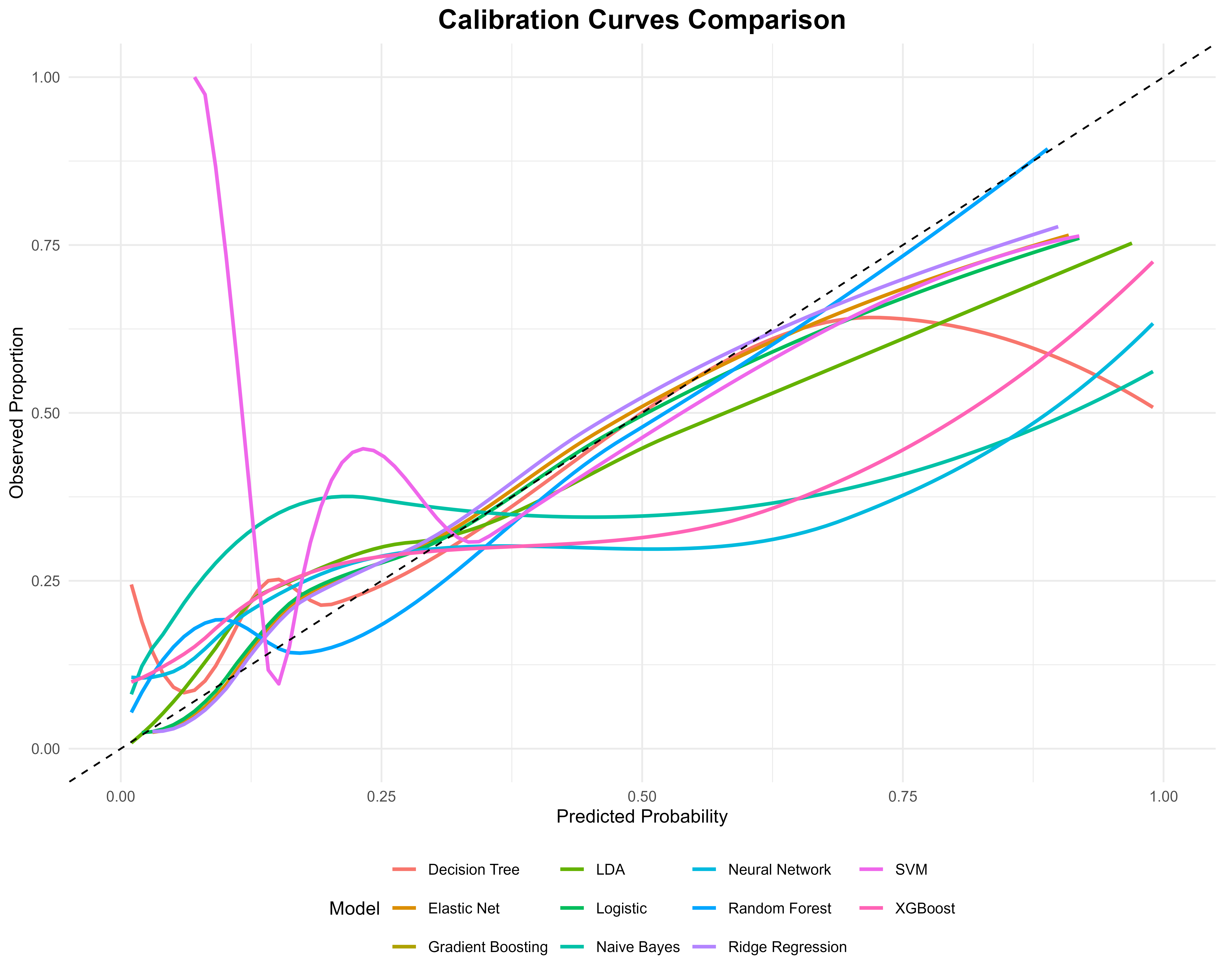


**Supplement Figure S2. Calibration curves of 11 machine learning models.**

Logistic regression, elastic net, and ridge regression showed the best calibration, closely following the ideal diagonal line across the entire probability range. LDA and Naive Bayes had moderate calibration, with slight under‑prediction at low probabilities and over‑prediction at high ones. SVM exhibited extreme miscalibration. Tree‑based models (random forest, XGBoost, decision tree) displayed sigmoid‑shaped curves with systematic bias. Neural network and gradient boosting performed poorly. The superior calibration of regularized linear models supports their clinical use for accurate risk estimation.


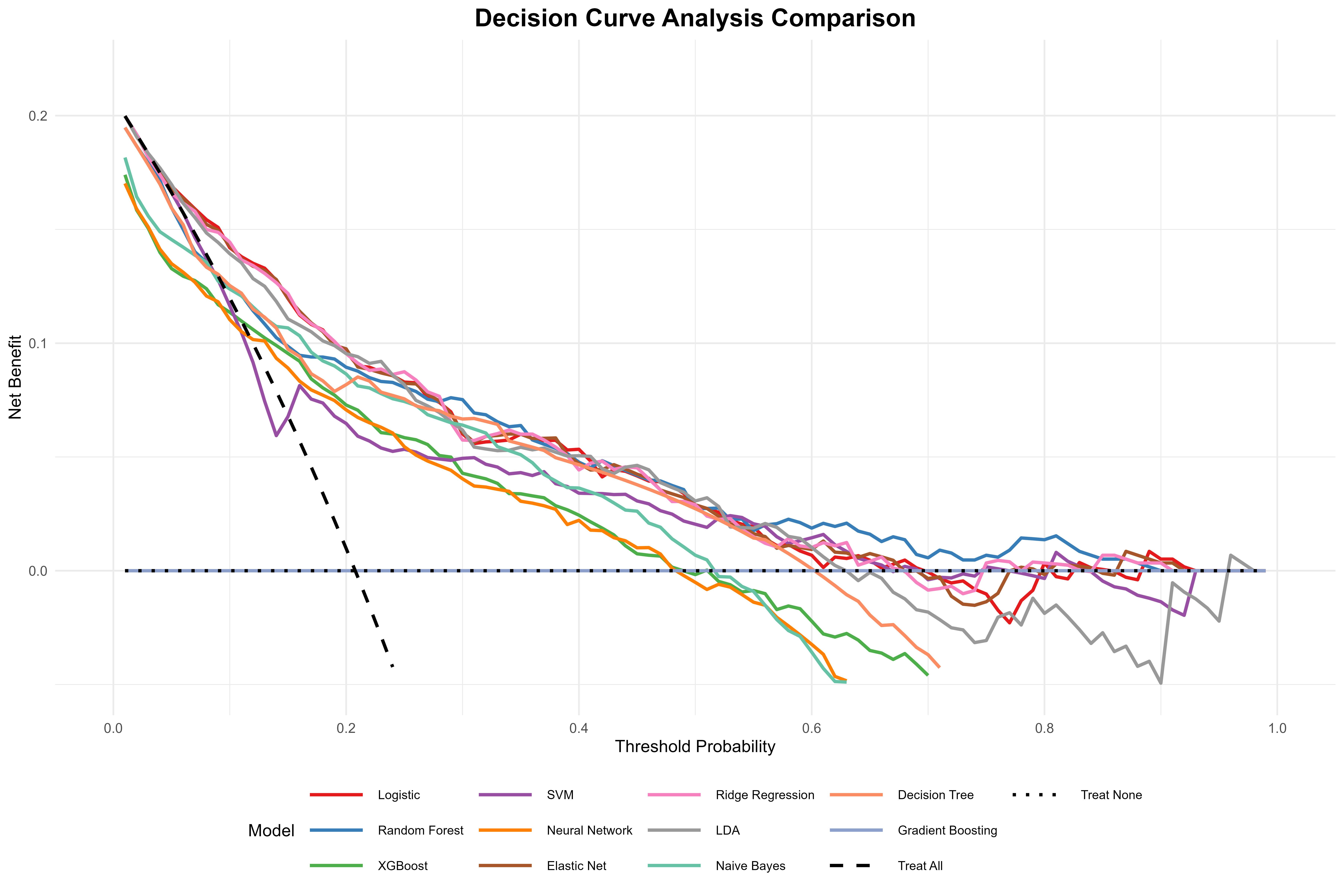
**Supplement Figure S3. Decision curve analysis of 11 machine learning models.**

Logistic regression, ridge regression, and elastic net provided the highest net benefit across the widest threshold range (approximately 0.05–0.75), consistently outperforming the “Treat All” and “Treat None” strategies. Naive Bayes and neural network showed moderate utility in the 0.1–0.6 range. SVM performed poorly, with negative net benefit below threshold 0.2. Tree‑based models (random forest, XGBoost, decision tree) exhibited erratic net benefit, often falling below zero at higher thresholds. LDA and gradient boosting had intermediate utility. The superior net benefit of regularized linear models supports their use for clinical decision‑making in ICU resource allocation and intervention.


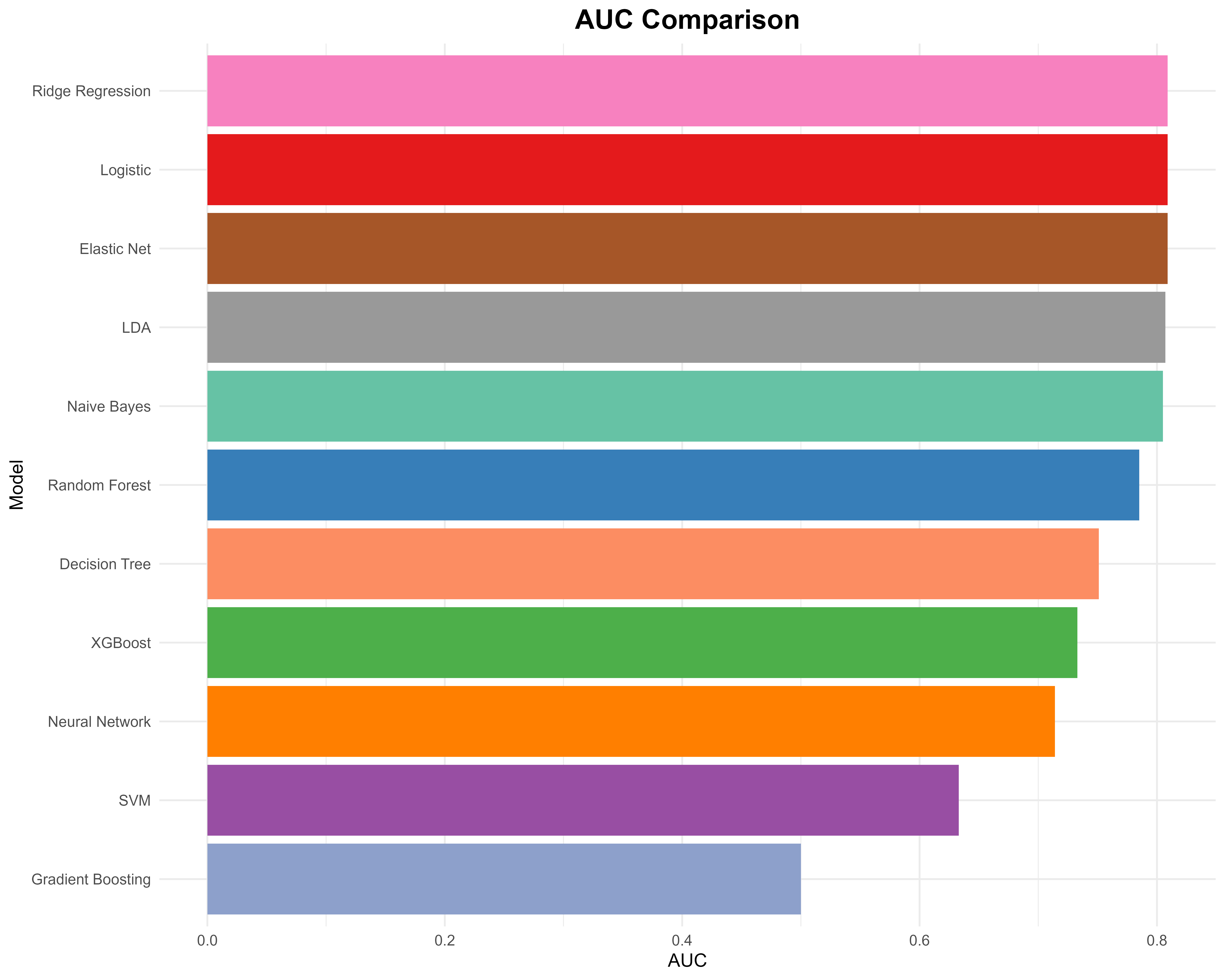
**Supplement Figure S4. Bar chart of AUC values across 11 models.**

Ridge regression, logistic regression, and elastic net achieved the highest AUC (≈0.809), followed by LDA (0.807) and Naive Bayes (0.805). Random forest (0.785) and decision tree (0.751) performed moderately. XGBoost (0.733), neural network (0.714), SVM (0.633), and gradient boosting (0.5) ranked lowest. The top three regularized linear models outperformed more complex algorithms, confirming that simplicity and appropriate regularization yield optimal discrimination for this prediction task.


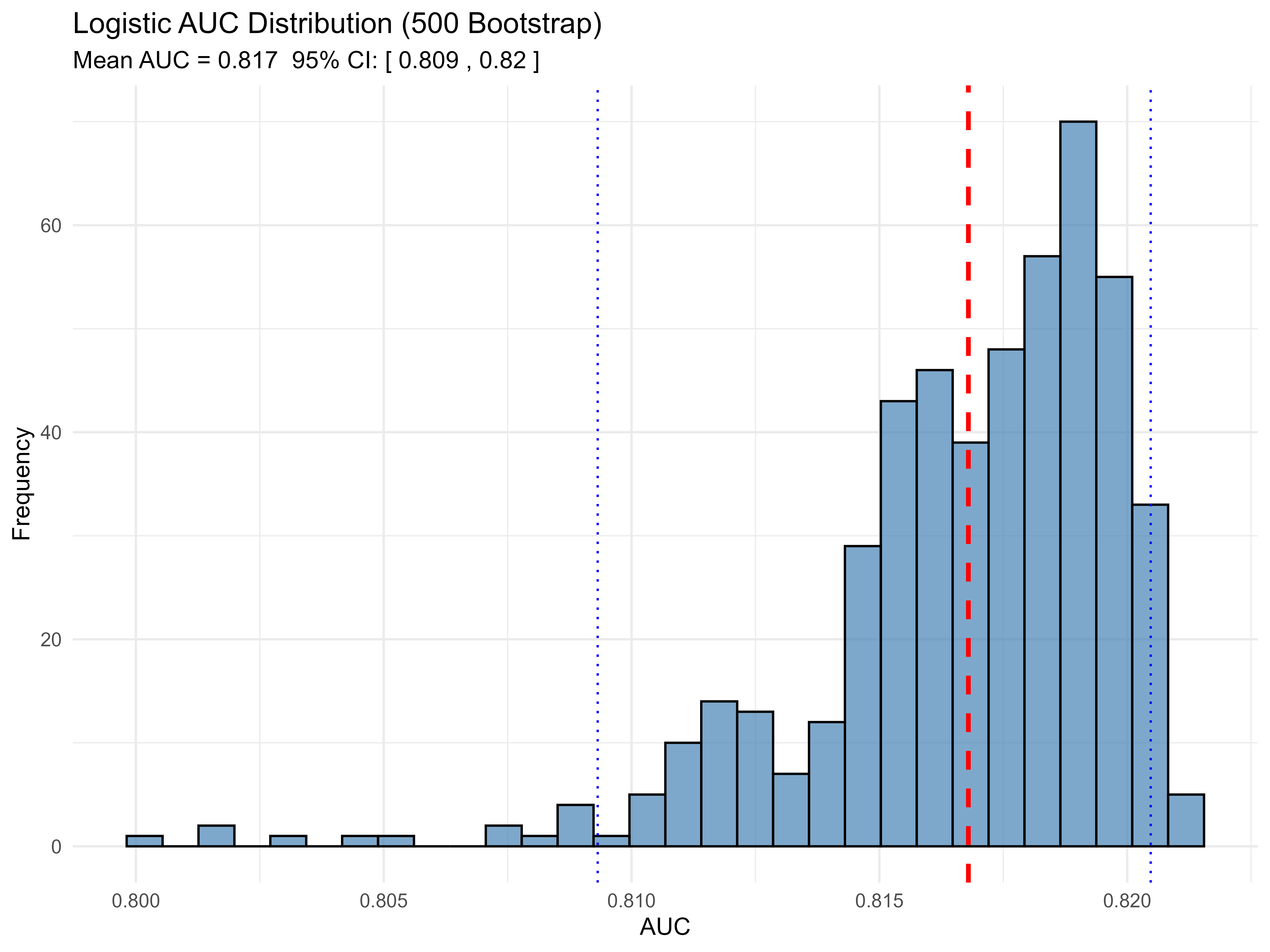
**Supplement Figure S5. Bootstrap distribution of AUC for logistic regression (500 iterations).**

The histogram shows AUC values ranging from 0.800 to 0.822, with a mean of 0.817 (95% CI: 0.809–0.820), closely approximating the original AUC (0.809). The narrow confidence interval and high concentration around the mean indicate robust model stability and minimal optimism bias, supporting generalizability.


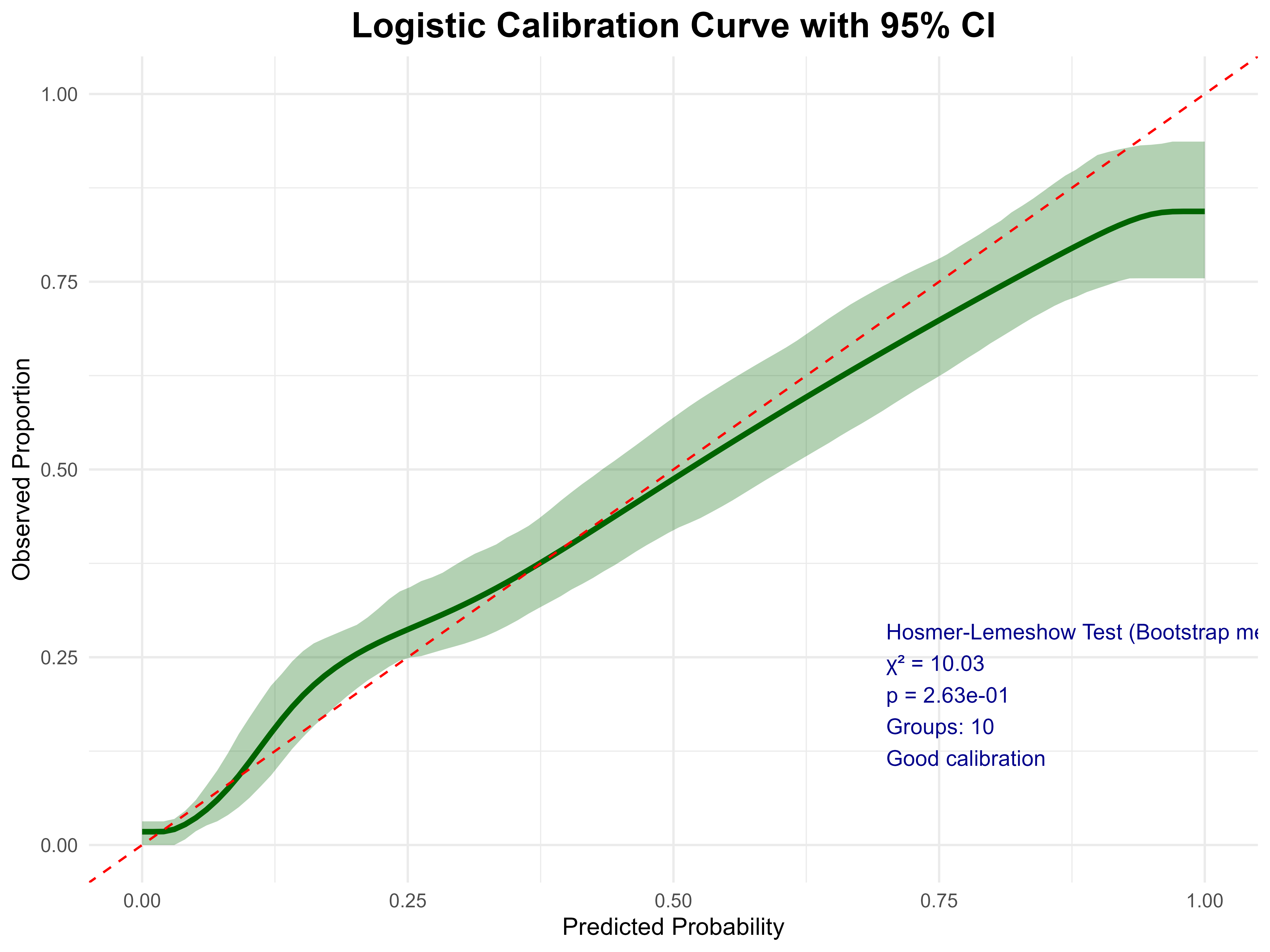
**Supplement Figure S6. Bootstrap-validated calibration curve for logistic regression (500 iterations).**

The smoothed calibration curve (green) closely follows the ideal diagonal line across the probability range 0–1.0, with the 95% confidence band (shaded) encompassing perfect calibration. Slight under‑prediction was observed at lower probabilities (0–0.25) and mild over‑prediction above 0.75, but the Hosmer‑Lemeshow test confirmed adequate calibration (χ² = 10.03, df = 8, P = 0.263). The model provides reliable probability estimates for clinical use.


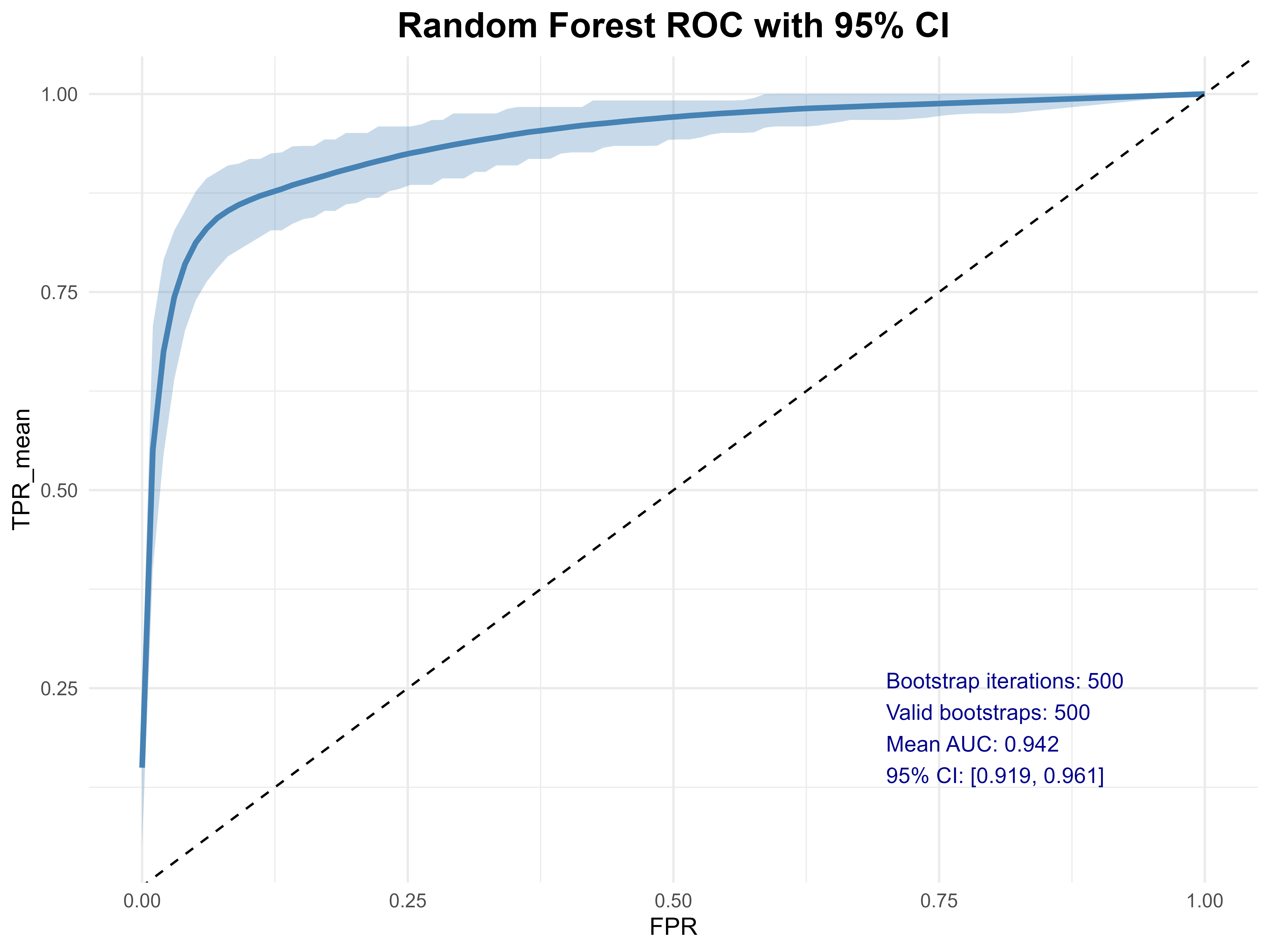
**Supplement Figure S7. Bootstrap-validated ROC curve for Random Forest (500 iterations).**

The mean AUC was 0.942 (95% CI: 0.919–0.961), substantially higher than the apparent AUC of 0.785 from the original analysis, suggesting overfitting. The 95% confidence band (shaded blue) shows moderate variability, particularly in the 0.1–0.3 false‑positive range. Despite excellent discriminative performance under bootstrap validation, calibration and clinical utility concerns (see Supplement Figures S2–S3) favored the selection of regularized linear models.


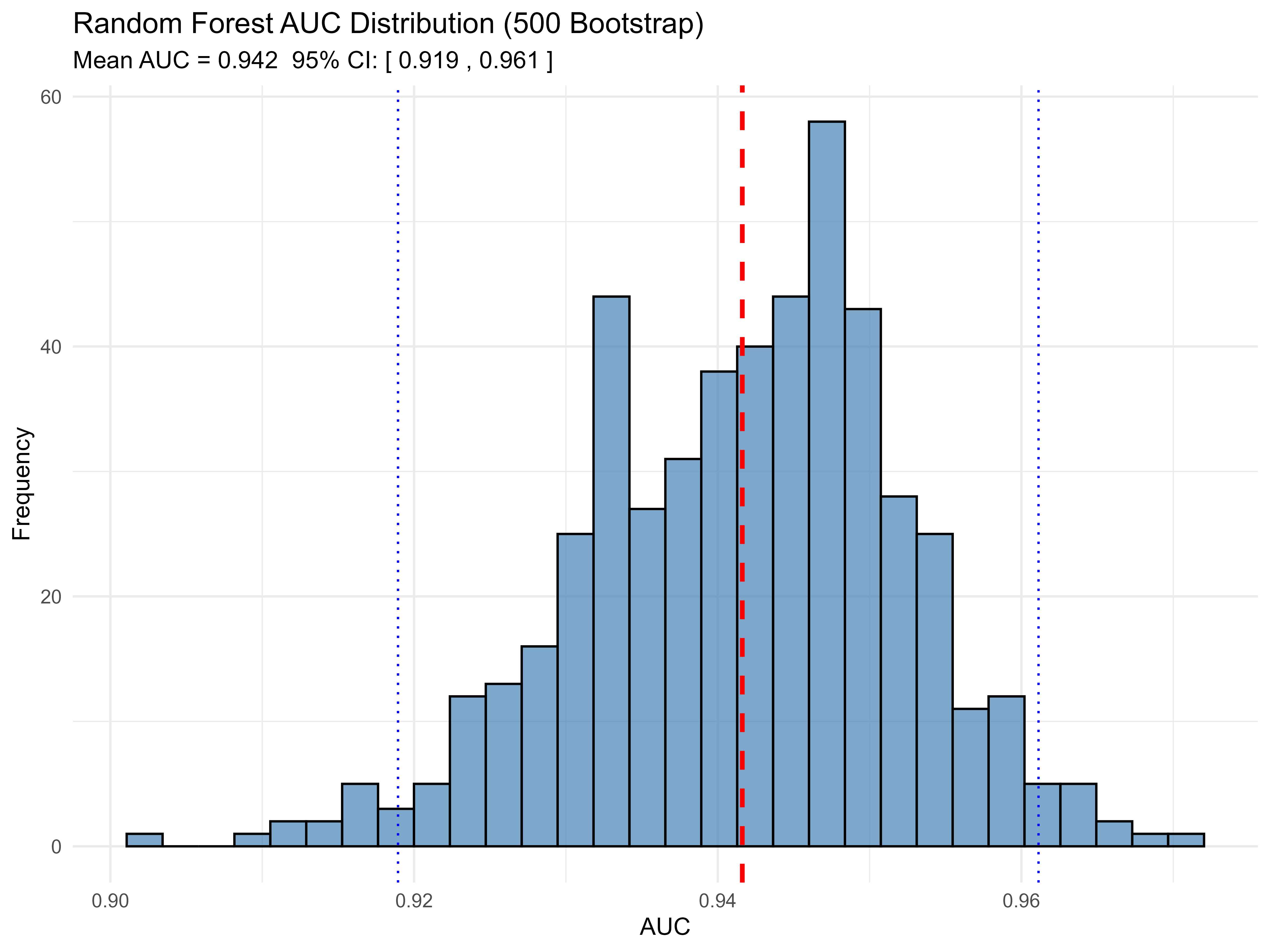
**Supplement Figure S8. Bootstrap distribution of AUC for Random Forest (500 iterations).**

The distribution is near‑symmetric, ranging from 0.90 to 0.97. Mean AUC = 0.942 (95% CI: 0.919–0.961), substantially higher than the apparent AUC of 0.785 from the original analysis, indicating overfitting. Despite high bootstrap AUC, poor calibration and net benefit (Supplement Figures S2–S3) favored logistic regression for clinical use.


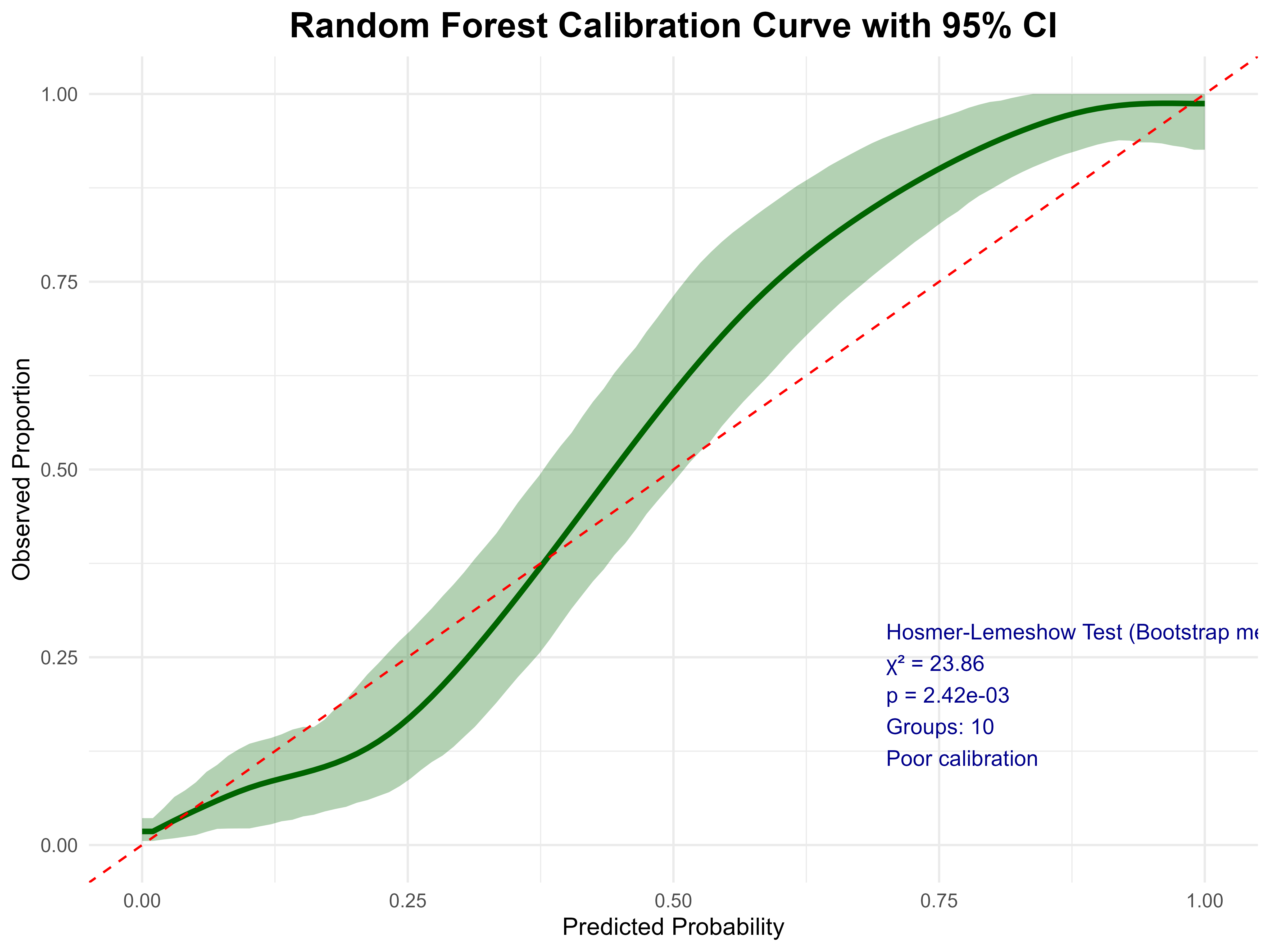
**Supplement Figure S9. Bootstrap-validated calibration curve for Random Forest (500 iterations).**

The calibration curve shows a pronounced sigmoid pattern, with under‑prediction at low probabilities and over‑prediction at high probabilities. The Hosmer‑Lemeshow test was significant (χ² = 23.86, df = 8, P = 0.0024), indicating poor calibration. Despite excellent discrimination (AUC = 0.942), the model’s unreliable probability estimates preclude clinical use, reinforcing the choice of logistic regression.


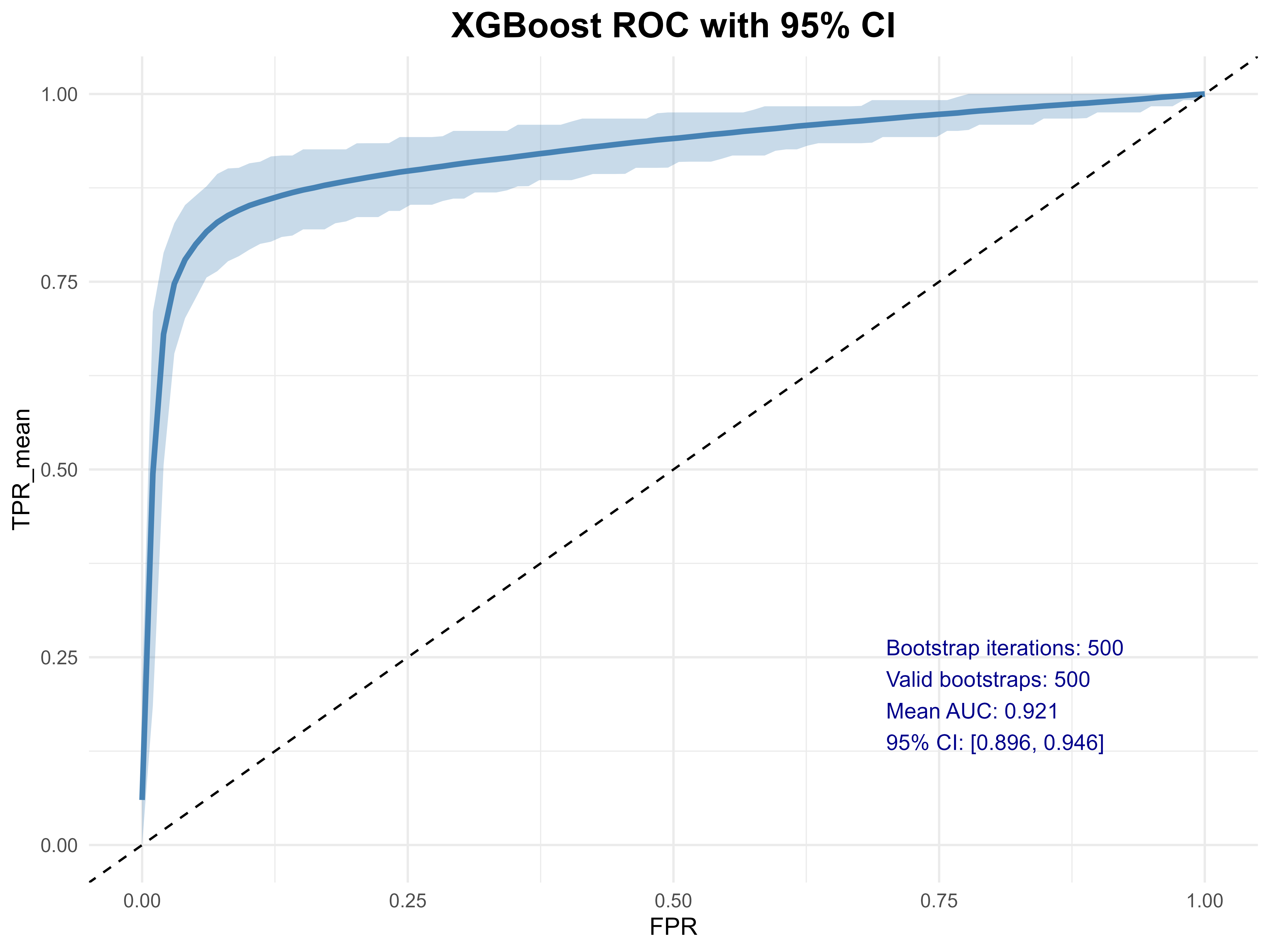
**Supplement Figure S10. Bootstrap-validated ROC curve for XGBoost (500 iterations).**

Mean AUC = 0.921 (95% CI: 0.896–0.946), substantially higher than the original AUC of 0.733, suggesting overfitting. The 95% confidence band (shaded blue) shows moderate variability at low false‑positive rates.


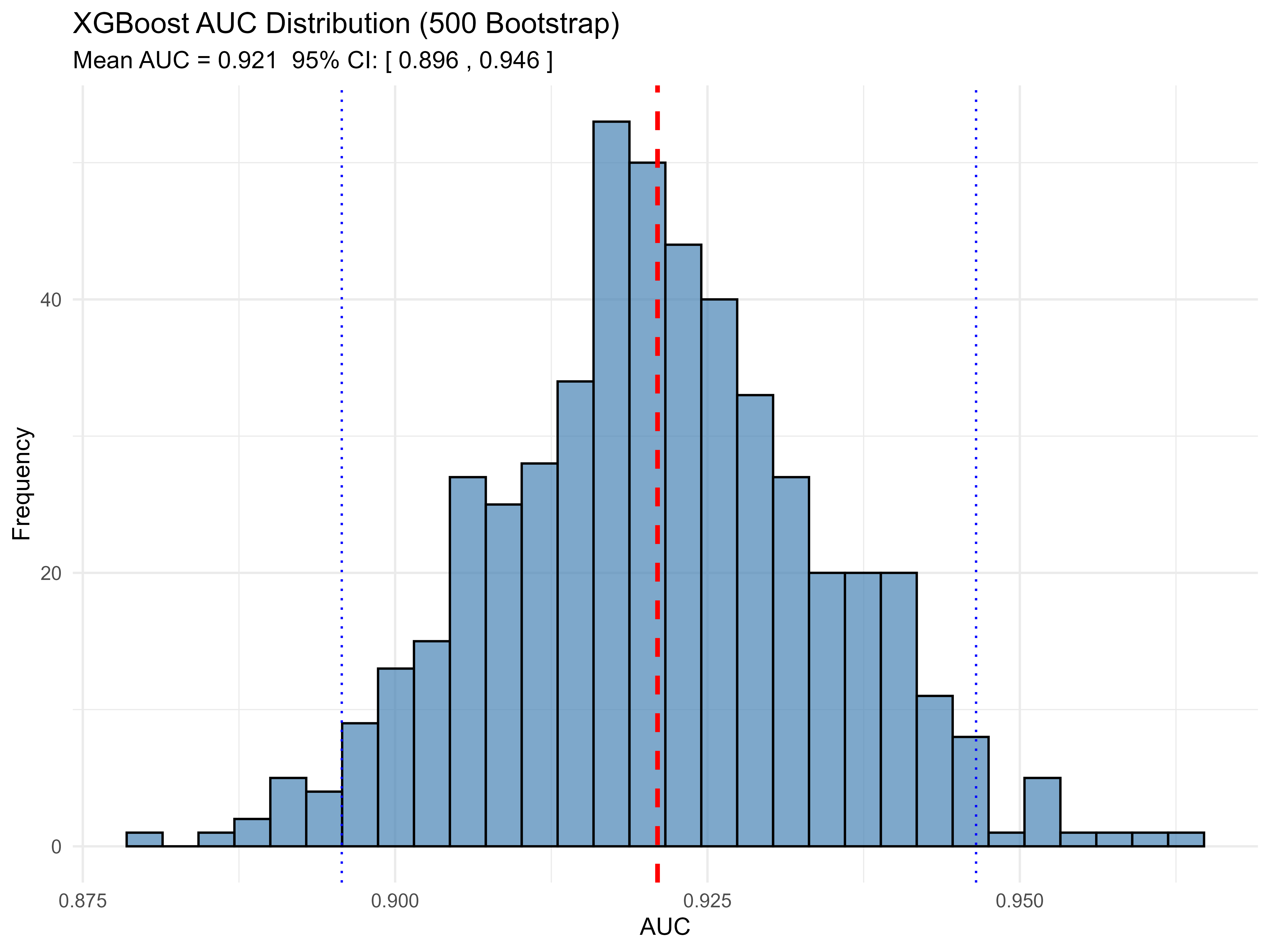
**Supplement Figure S11. Bootstrap distribution of AUC for XGBoost (500 iterations).**

The distribution is approximately normal, ranging from 0.875 to 0.955. Mean AUC = 0.921 (95% CI: 0.896–0.946), exceeding the original AUC (0.733), indicating optimism bias. Poor calibration and net benefit (Supplement Figures S2–S3) favored logistic regression.


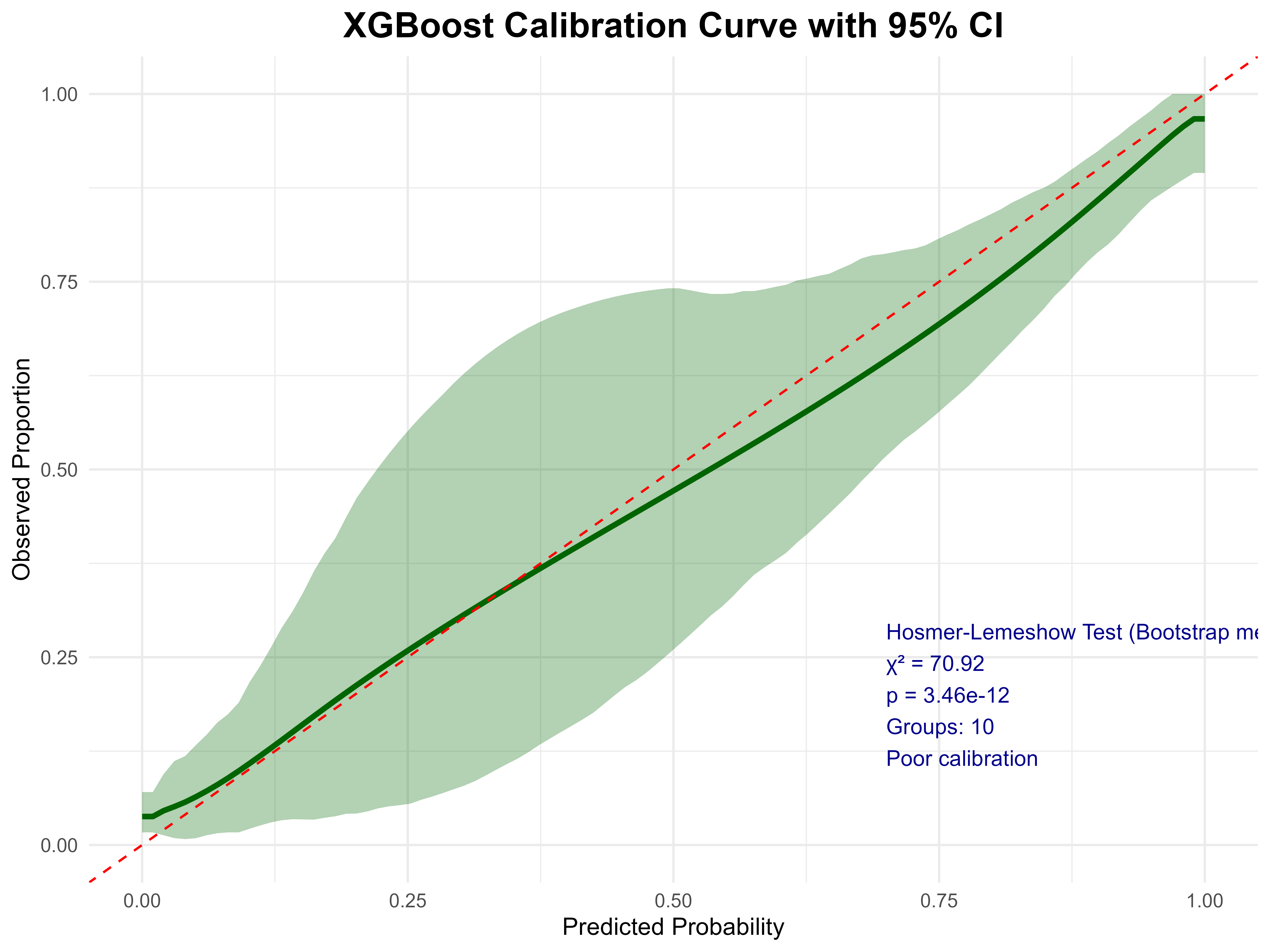
**Supplement Figure S12. Bootstrap-validated calibration curve for XGBoost (500 iterations).**

The curve shows severe sigmoid miscalibration: under‑prediction at low‑to‑moderate probabilities and over‑prediction at higher ranges. Hosmer‑Lemeshow test: χ² = 70.92, df = 8, P = 3.46 × 10⁻¹², indicating extremely poor calibration despite high AUC.


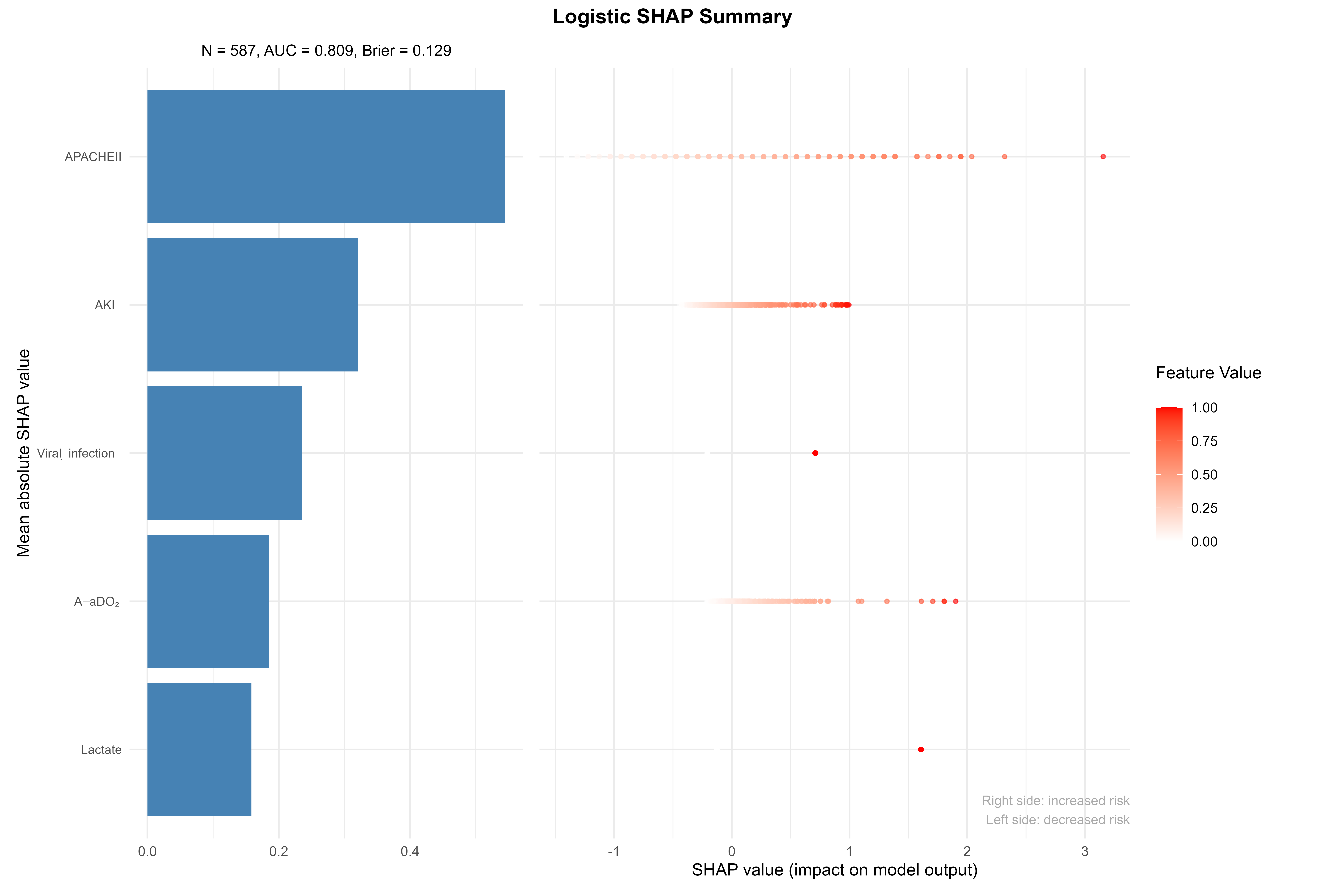
**Supplement Figure S13. SHAP summary plot for logistic regression.**

Left panel: mean absolute SHAP values rank APACHE II highest, followed by AKI, viral infection, A‑aDO₂, and lactate. Right panel: SHAP value distribution shows direction of impact (right = increased risk). APACHE II shows a continuous gradient; AKI and viral infection binary risk elevation; A‑aDO₂ and lactate predominantly risk‑increasing.


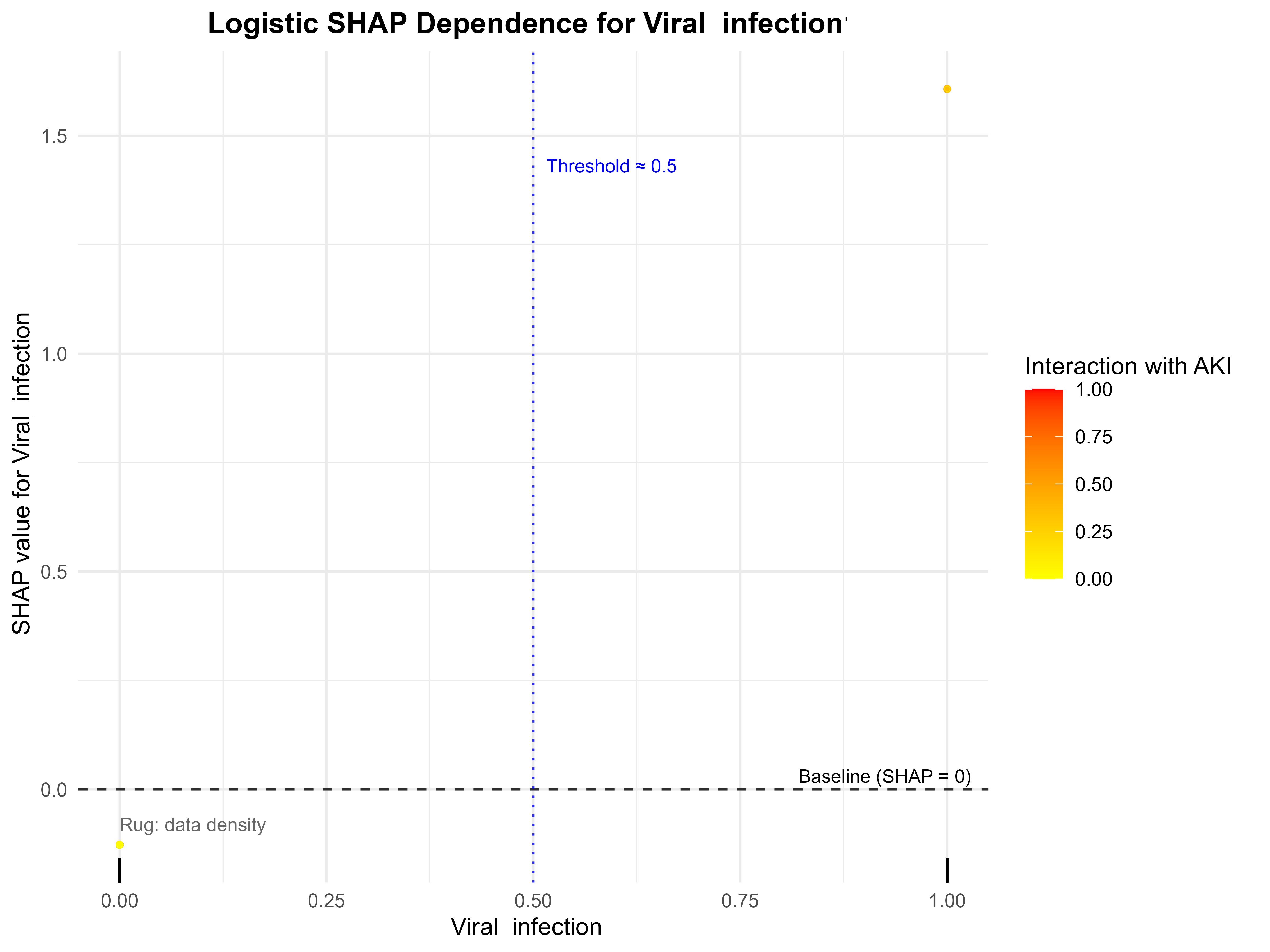
**Supplement Figure S14. SHAP dependence plot for viral infection (interaction with AKI).**

Viral infection presence (x = 1) sharply increases SHAP from 0 to ≈1.6. The highest SHAP values occur when AKI is absent, suggesting independent risk. Most patients have no viral infection.


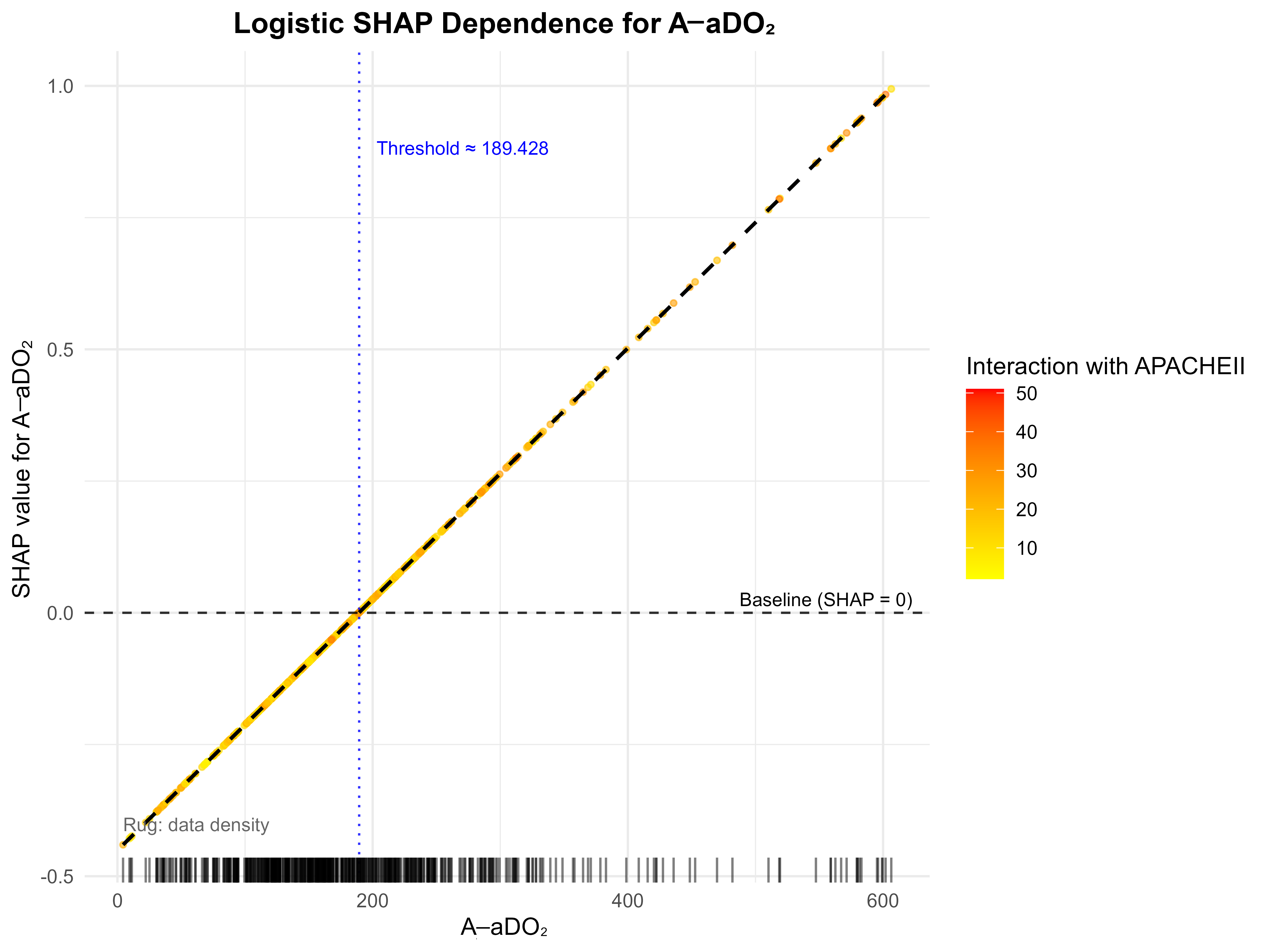
**Supplement Figure S15. SHAP dependence plot for A‑aDO₂ (interaction with APACHE II).**

Near‑linear positive association: SHAP increases from ≈‑0.4 (A‑aDO₂ ≈ 0) to ≈1.0 (600 mmHg). Threshold at 189.4 mmHg marks transition to positive risk. Higher APACHE II co‑occurs across the range, indicating complementary information.


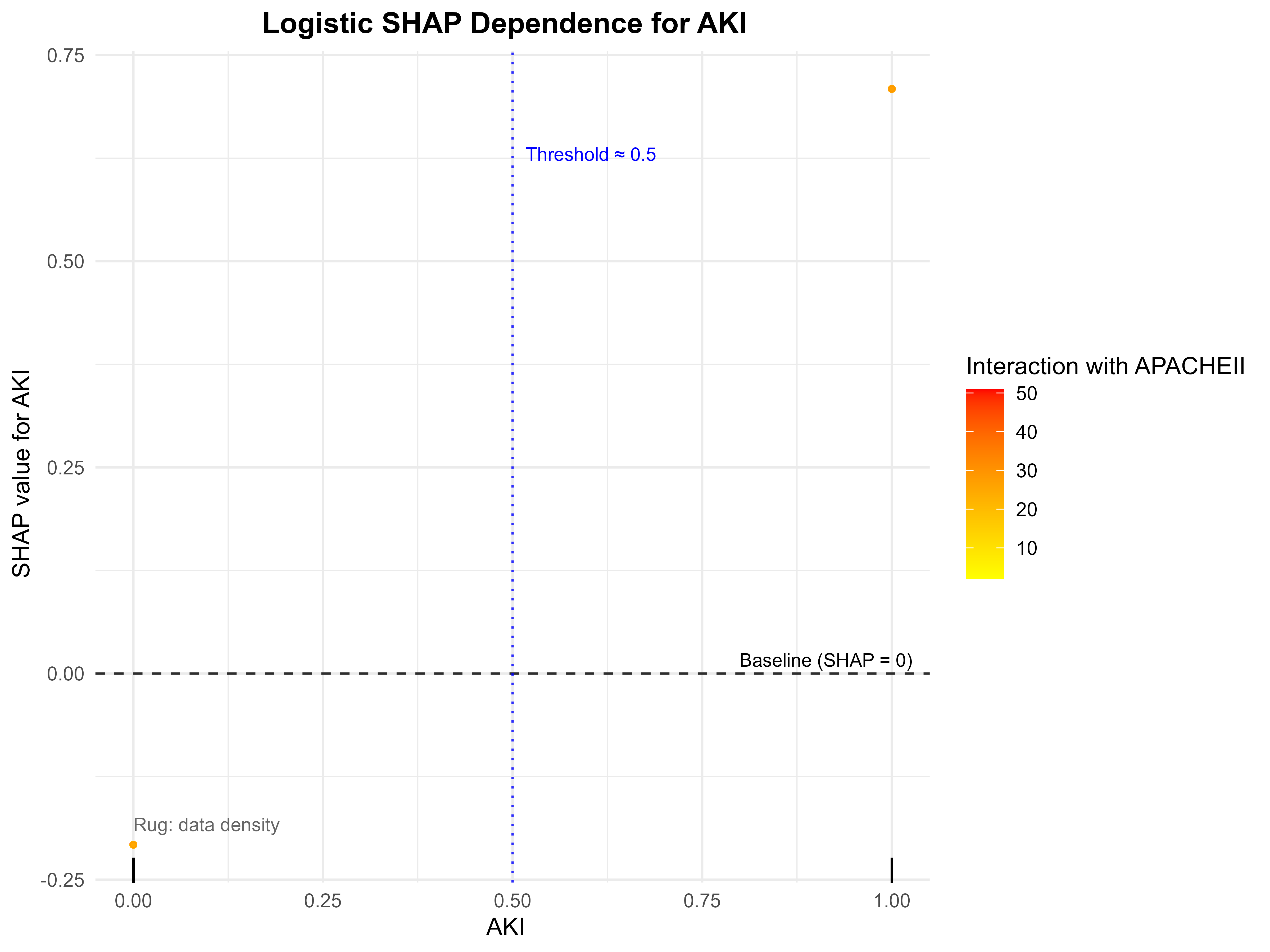
**Supplement Figure S16. SHAP dependence plot for AKI (interaction with APACHE II).**

Binary threshold at 0.5: AKI absence gives SHAP ≈ ‑0.2; presence increases SHAP to ≈0.7. Highest SHAP occurs with lower APACHE II, suggesting AKI adds independent risk. Most patients have no AKI.


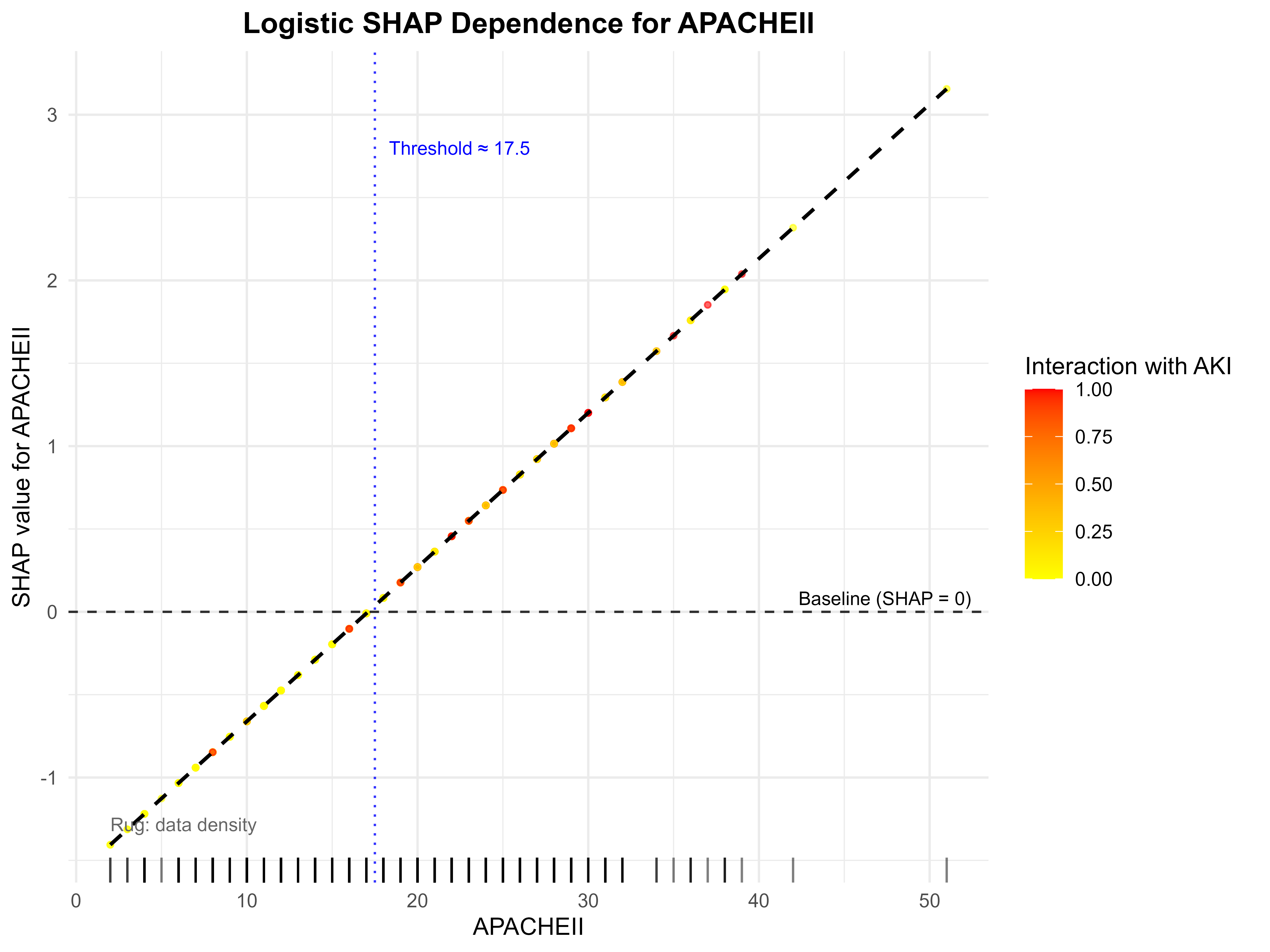
**Supplement Figure S17. SHAP dependence plot for APACHE II (interaction with AKI).**

Strong positive linear relationship: SHAP from ≈‑1.2 (APACHE II ≈ 0) to ≈3.2 (≈55). Threshold at 17.5 marks transition to harmful effect. Higher APACHE II co‑occurs with AKI above threshold, indicating synergy.


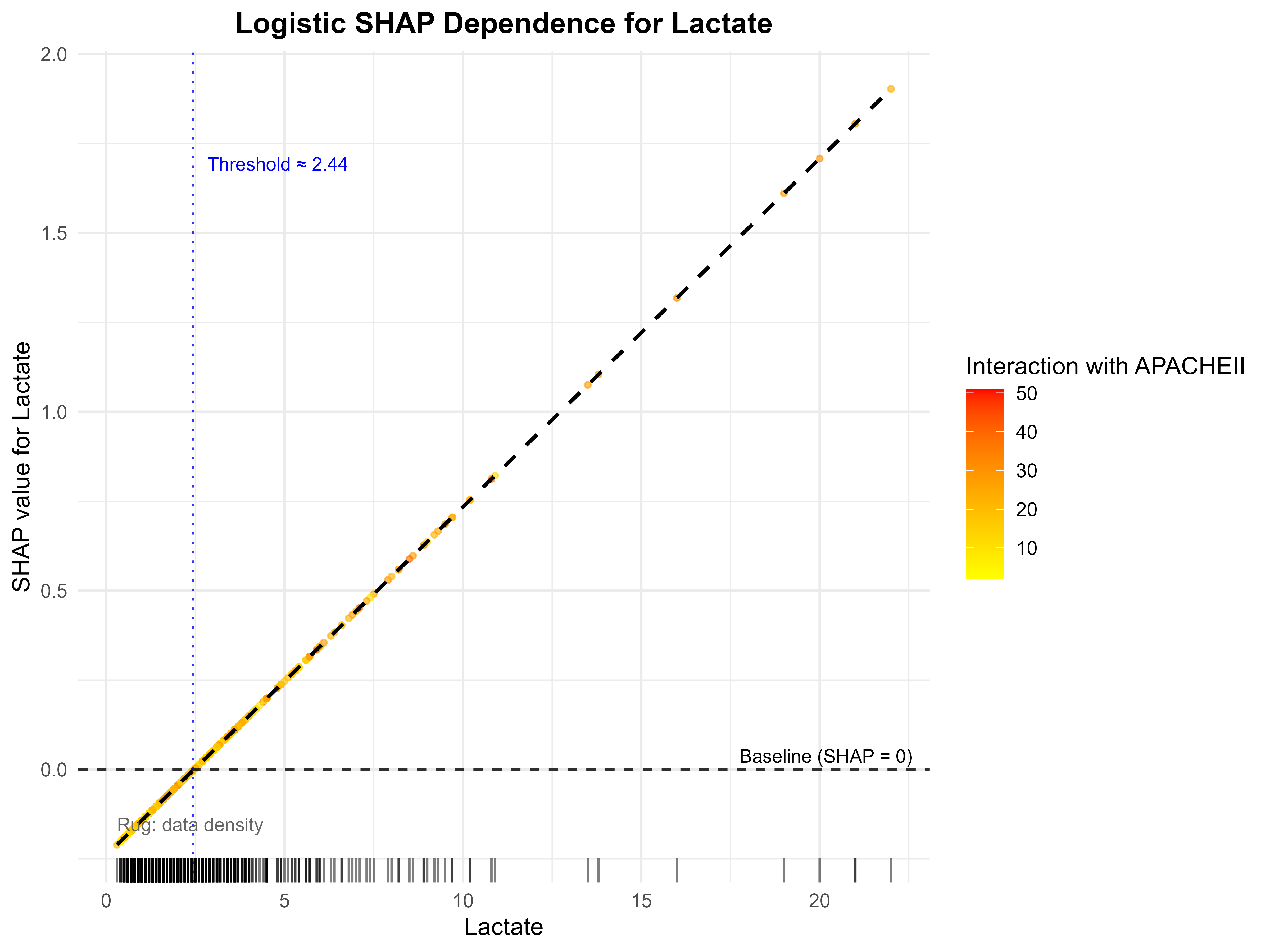
**Supplement Figure S18. SHAP dependence plot for lactate (interaction with APACHE II).**

Positive linear trend: SHAP from ≈‑0.2 (lactate ≈ 0) to ≈1.9 (≈22 mmol/L). Threshold at 2.44 mmol/L. Higher lactate co‑occurs with higher APACHE II above threshold, consistent with hypoperfusion pathophysiology.
